# Molecular identification, genotyping and phylogenetic analysis of *Ixodes* and *Rhipicephalus* ticks and their associated spotted fever group *Rickettsia* species from a single location in northern Tunisia

**DOI:** 10.3389/fmicb.2025.1644524

**Published:** 2025-08-14

**Authors:** Myriam Kratou, Hanène Belkahia, Rachid Selmi, Meriam Ben Abdallah, Ghassan Tayh, Lilia Messadi, Mourad Ben Said

**Affiliations:** ^1^Laboratory of Microbiology, National School of Veterinary Medicine of Sidi Thabet, University of Manouba, Manouba, Tunisia; ^2^Ministry of National Defense, General Directorate of Military Health, Veterinary Service, Tunis, Tunisia; ^3^Department of Basic Sciences, Higher Institute of Biotechnology of Sidi Thabet, University of Manouba, Manouba, Tunisia

**Keywords:** vector-borne rickettsioses, ticks, *Ixodes* genus, *Rhipicephalus sanguineus* sensu lato, molecular identification, genetic diversity, phylogeny, Tunisia

## Abstract

**Introduction:**

Ticks and their associated spotted fever group *Rickettsia* (SFGR) represent an emerging zoonotic risk in Tunisia, where data on tick species distribution and pathogen prevalence remain limited. This study specifically aimed to investigate the diversity and phylogeny of *Ixodes* and *Rhipicephalus* tick species and to identify and genetically characterize their associated SFGR species in northwestern Tunisia.

**Methods:**

Tick sampling was conducted over a five-month period, from November 2022 to March 2023, in the Jouza district, Beja Governorate, northern Tunisia. A total of 236 ticks were collected both from vegetation using flag-dragging and manually from a red fox (*Vulpes vulpes*) carcass encountered opportunistically in the field. Tick species were morphologically identified and confirmed by Sanger sequencing of the mitochondrial 16S rRNA gene. *Rickettsia* detection was performed by nested PCR targeting the *ompB* gene, followed by species-level identification through sequencing of *ompA* and *gltA* partial sequences. Phylogenetic analyses were conducted to assess genetic relationships. Additionally, chi-square tests were used to assess differences in infection rates between tick species, life stages, and collection sources.

**Results:**

Ticks belonged to the *Ixodes ricinus* complex (*Ix. ricinus* and *Ix. inopinatus*), *Ix. hexagonus*, and the *Rhipicephalus sanguineus* sensu lato complex (*Rh. sanguineus* sensu strict and *Rh. rutilus*) have been identified. Twelve and ten genotypes were recorded from 45 and 59 partial 16S rRNA mitochondrial sequences isolated from *Ix. ricinus* and *Ix. inopinatus*, respectively. Additionally, one genotype was recorded from five *Rh. rutilus* specimens, and four genotypes were detected among 23 *Rh. sanguineus* (temperate lineage) individuals. Overall, 52.1% of ticks tested positive for *Rickettsia* spp., with significantly higher infection rates in *Ix. ricinus* (75.6%, *p* < 0.001) and *Ix. inopinatus* (67.4%, *p* < 0.01) compared to the *Rhipicephalus* group (37.2%) and *Ix. hexagonus*, which tested negative. Three *Rickettsia* species were identified: *R. monacensis* and *R. helvetica* in the *Ix. ricinus* complex (including one co-infection), and *R. massiliae* exclusively in *Rhipicephalus* ticks. Moreover, phylogenetic analysis revealed that our tick isolates and associated *Rickettsia* spp. from questing ticks and the red fox clustered primarily with those from other North African and Southern European countries, suggesting trans-Mediterranean strain circulation and a potential link to wildlife reservoirs.

**Conclusion:**

These findings enhance our understanding of tick and SFGR diversity in Tunisia and underscores the zoonotic risks from co-circulating *Rickettsia* species in shared environments. The high infection rates in *Ix. ricinus* and *Ix. inopinatus* call for improved national tick surveillance. Public health implications include the need to consider tick-borne rickettsioses in unexplained febrile illness diagnoses. Future studies should assess infection status in hosts, explore seasonal tick dynamics, and evaluate environmental factors affecting *Rickettsia* transmission.

## 1 Introduction

Ticks are obligate hematophagous ectoparasites and prominent vectors of a wide range of pathogens affecting human and animal health globally (Nováková and Šmajs, 2019). Environmental changes including climate shifts, land-use transformation, and altered wildlife dynamics have facilitated the geographic expansion of ticks and their associated pathogens, particularly in the Mediterranean basin (Estrada-Peña and de la Fuente, 2014; Martina et al., 2017; Sonenshine, 2018). Among tick families, Ixodidae is the most epidemiologically relevant, owing to its abundance, ecological versatility, and broad host range. These characteristics position it centrally within the *One Health* framework (Tsatsaris et al., 2016).

Within this context, tick-borne zoonoses, especially vector-borne rickettsioses (VBRs), have gained increasing public health attention (Onyiche et al., 2022). VBRs are caused by obligate intracellular Gram-negative bacteria of the genus *Rickettsia*, which are primarily transmitted by arthropods, with hard ticks (Acari: Ixodidae) acting as both vectors and reservoirs (Parola et al., 2005, 2013). In addition, over 27 of the 30 recognized *Rickettsia* species are associated with hard ticks, and the majority belong to the spotted fever group (SFG), including *R. rickettsii* and *R. conorii*, the causative agents of Rocky Mountain spotted fever and Mediterranean spotted fever, respectively (Parola et al., 2005). These pathogens exhibit diverse vector-host associations and pathogenic profiles, resulting in variable clinical outcomes (Kernif et al., 2012). In Africa, at least 17 *Rickettsia* species have been identified across several tick genera, including *Hyalomma, Amblyomma, Rhipicephalus*, and *Ixodes*, underscoring the ecological complexity and zoonotic potential of SFG rickettsioses (Parola et al., 2013). These bacteria are primarily maintained in tick populations through vertical transmission (transovarial and transstadial), although horizontal transmission *via* vertebrate hosts also plays a critical role (Hauck et al., 2020; Laukaitis and Macaluso, 2021). However, some *Rickettsia* spp. negatively impact tick fitness by reducing fecundity and reproductive success (Nieri-Bastos et al., 2013; Krawczak et al., 2016; Gerardi et al., 2019). In addition, tick distribution is strongly influenced by abiotic factors, particularly temperature and humidity, which in turn affect their pathogen transmission potential (Onyiche et al., 2022).

Despite growing awareness, the epidemiological landscape of SFG *Rickettsia* in North Africa remains insufficiently documented. In Tunisia, *Rickettsia* infections have been documented for over a century, beginning with *R. conorii*, the causative agent of Mediterranean spotted fever, first reported in 1910 (Znazen et al., 2013). Nonetheless, more recent molecular studies have revealed a broader range of circulating species, including *R. helvetica, R. africae, R. aeschlimannii*, and *R. massiliae*, primarily detected in *Hyalomma* and *Rhipicephalus* ticks from domestic animals in central, southern and northern Tunisia (Demoncheaux et al., 2012; Khrouf et al., 2013; Selmi et al., 2019; Belkahia et al., 2021; Kratou et al., 2023). In this context, attention is increasingly turning to the role of wildlife hosts in pathogen maintenance and transmission. Case in point, red foxes (*Vulpes vulpes*) are becoming increasingly recognized as important hosts in the ecology of tick-borne pathogens, due to their wide distribution, synanthropic behavior, and frequent infestation by diverse tick species (Hornok et al., 2017; Chisu et al., 2014). As potential bridge hosts between wildlife and peri-domestic environments, they serve as valuable sentinels for monitoring the circulation of zoonotic agents such as *Rickettsia* (Marié et al., 2011; Liu et al., 2021; Selmi et al., 2025). However, in Tunisia, data on the tick fauna associated with red foxes and their infection status remain scarce, limiting our understanding of their role in pathogen maintenance and transmission.

Furthermore, the northwestern region of Tunisia, particularly the Jouza district in the Beja Governorate, provides a humid, forested habitat that supports diverse tick populations, including the sympatric occurrence of *Ixodes ricinus* and *Ixodes inopinatus*, both competent vectors of zoonotic pathogens (Zhioua et al., 1994; Sarih et al., 2003; Younsi et al., 2005; Selmi et al., 2024). In parallel, *Rhipicephalus sanguineus* sensu lato is the most widespread tick species in the Mediterranean basin and a primary vector of *R. conorii*, the causative agent of Mediterranean spotted fever (Kim, 2022). Within this complex, *Rh. sanguineus* sensu stricto and *Rh. rutilus* are key vectors of several zoonotic pathogens, including *R. conorii, R. massiliae, R. felis, Ehrlichia canis*, and *Coxiella burnetii* (Dantas-Torres, 2010; Khrouf et al., 2013; Šlapeta et al., 2023; Senbill et al., 2024).

Despite increasing reports of *Rickettsia* species in Tunisia, data on the genetic diversity and infection status of *Ixodes* and *Rhipicephalus* ticks with spotted fever group (SFG) *Rickettsia* remain limited. Given the ecological importance of both questing and host-associated ticks in pathogen transmission, further investigation is warranted, particularly regarding wildlife hosts such as the red fox (*V. vulpes*) as potential reservoirs. This study is an exploratory investigation focused on a single location in a sub-humid zone in northern Tunisia, known for its high infestation primarily by *Ixodes* ticks. In particular, we aimed to characterize the species composition, genetic diversity, and phylogenetic relationships of *Ixodes* and *Rhipicephalus* ticks collected from vegetation and an accidentally found red fox during sampling, and their associated SFG *Rickettsia* spp. Additionally, infection prevalence was assessed across tick species, life stages, and collection sources. We hypothesized that both questing and host-associated ticks in this region harbor a genetically diverse pool of SFG *Rickettsia*, with red foxes potentially playing a role in their maintenance and transmission within wildlife-associated cycles.

## 2 Materials and methods

### 2.1 Study regions, tick collection and morphological identification

**Figure 1:**
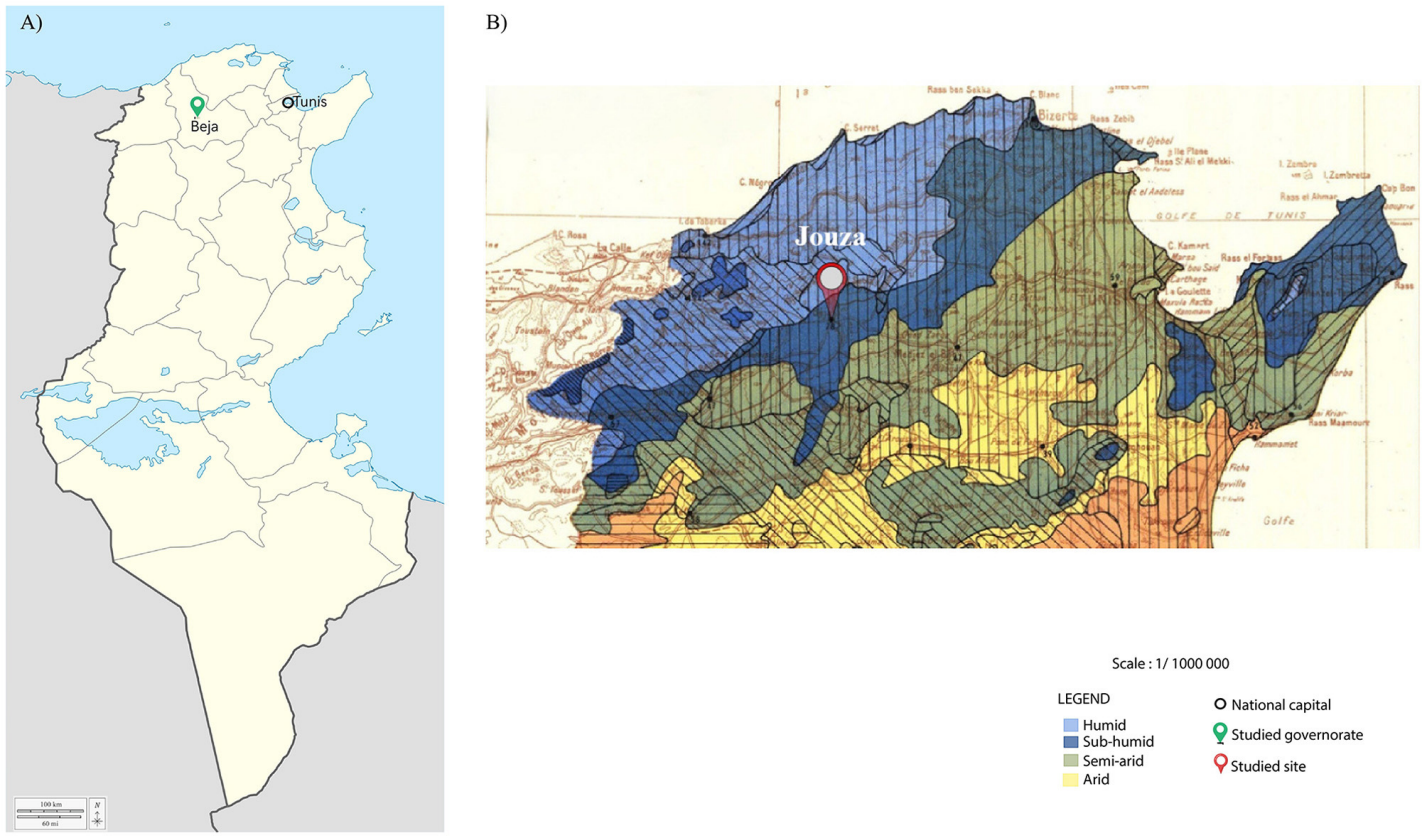
Maps of the study area in Tunisia. (A) Map of Tunisia divided by governorates, showing the location of the Beja governorate marked with a green pin and the national capital, Tunis, indicated by a black circle. (B) Zoomed-in map of northwestern Tunisia based on bioclimatic stages, showing the specific sampling site in the Jouza district marked with a red pin. National borders are outlined in black.

Between November 2022 and March 2023, a total of 236 ticks were collected from a single site in the Jouza district, Beja Governorate, northwestern Tunisia, characterized by a humid and subhumid bioclimatic stage favorable to the presence of *Ixodes* and *Rhipicephalus* species (Figure 1). Tick sampling targeted this site specifically due to its known ecological suitability for these genera. Ticks were collected from both vegetation and a red fox (*V. vulpes*). Vegetation sampling was performed using the standard flag-dragging technique, involving sweeping a white flannel cloth over the vegetation to capture questing ticks. Ticks from the red fox were collected manually. The fox, found dead by chance on a rural road in the Jouza region, was a single adult individual that appeared to have died recently. Remarkably, it was heavily infested with *Ixodes* ticks, an original and noteworthy finding as investigations on red fox tick infestations in this region have not previously been conducted. Ticks were carefully removed using forceps from preferred attachment sites on the fox's body, including the ears, neck, udder area, and external genitalia. Since only this single carcass was sampled, the risk of resampling the same animal was eliminated. All collected ticks were placed in sterile, labeled tubes and preserved individually in 70% ethanol, then stored at −20 °C until further analysis. Morphological identification was performed using standard taxonomic keys (Walker et al., 2005; Estrada-Peña et al., 2017), allowing classification by species, life stage, and sex.

### 2.2 Extraction of total DNA and amplification of tick DNA

Each tick specimen was washed three times in sterile water with gentle vortexing (10 s each), briefly rinsed in 70% ethanol, air-dried, and then individually homogenized using an automated TissueLyser LT system (Qiagen, Hilden, Germany). Genomic DNA was extracted using the DNeasy Tissue Kit (Qiagen), following the manufacturer's protocol, and stored at −20 °C until further use. To evaluate the efficiency of DNA extraction, PCR amplification of the mitochondrial 16S rRNA gene was performed using tick-specific primers TQ16S+1F and TQ16S-2R, as described by (Black and Piesman 1994) (Table 1). Each PCR run included both positive and negative controls to ensure the reliability and specificity of amplification.

**Table 1:** Primers used for the identification and/or genetic characterization of *Rickettsia* species infecting ticks collected in this study.

**Assays**	**Target genes**	**Primers**	**Sequences (5'-3')**	**Amplicon size (bp)**	**References**
**Single PCR** ^a^					
	16S rRNA	TQ16S+1F	CTGCTCAATGATTTTTTAAATTGCTGTGG	324	(Black and Piesman, 1994)
		TQ16S-2R	ACGCTGTTATCCCTAGAG		
**Nested PCR** ^b^					
First PCR	*ompB*	rompB_OF	GTAACCGGAAGTAATCGTTTCGTAA	511	(Choi et al., 2005)
		rompB OR	GCTTTATAACCAGCTAAACCACC		
Second PCR		rompB_SFG_IF	GTTTAATACGTGCTGCTAACCAA	425	
		rompB SFG-IR	GGTTTGGCCCATATACCATAAG		
**Semi-nested PCR** ^c^					
First PCR	*ompA*	Rr190.70p	ATGGCGAATATTTCTCCAAAA	631	(Oteo et al., 2006)
		Rr190.701n	GTTCCGTTAATGGCAGCATCT		
Second PCR		Rr190.70p	ATGGCGAATATTTCTCCAAAA	532	
		Rr190.602n	AGTGCAGCATTCGCTCCCCCT		
**Single PCR** ^c^					
	*gltA*	RpCS.877p	GGGGGCCTGCTCACGGCGG	381	(Regnery et al., 1992)
		RpCS.1258n	ATTGCAAAAAGTACAGTGAACA		

^a^Single PCR based on 16S rRNA gene allowing the selection of tick samples with DNA extraction efficiency.

^b^Nested PCR based on *ompB* gene allowing the detection and/or the characterization after sequencing of *Rickettsia* species.

^c^Single and Semi-nested PCR based on *gltA* and *ompA* genes, respectively, allowing the characterization after sequencing of *Rickettsia* species.

### 2.3 Identification of *Rickettsia* species

Initially, a nested PCR was conducted to amplify a 425 bp fragment of the rickettsial outer membrane protein B (*ompB*) gene from tick DNA samples, in order to detect *Rickettsia* species. Subsequently, single PCRs were used for species-level characterization amplifying gene fragments encoding the outer membrane protein A (*ompA*) and citrate synthase protein (*gltA*), measuring 532 bp and 381 bp, respectively. All PCR assays were performed using an automated DNA thermal cycler, following the thermal cycling protocols established by (Oteo et al. 2006) and (Regnery et al. 1992). PCR reactions were carried out in a total volume of 50 μL, containing 0.125 U/μL of Taq DNA polymerase (Biobasic Inc., Markham, Canada), 1x PCR buffer, 0.2 mM of dNTPs, 1.5 mM of MgCl_2_, 3 μL of genomic DNA (50–150 ng) for the first PCR, and 1 μL for the nested PCR. Additionally, 0.5 μM of primers and autoclaved water were included. Each PCR run included both positive and negative controls to ensure the reliability and specificity of amplification. PCR products were subsequently analyzed using electrophoresis on 1.5% agarose gels stained with ethidium bromide, allowing visualization under UV transillumination.

### 2.4 Statistical analysis

Confidence intervals (CIs) for prevalence rates were calculated at the 95% confidence level. To investigate the association between tick-related factors, including species and gender, on the molecular prevalence of *Rickettsia* species, either a chi-square test or Fisher's exact test were conducted. These analyses were performed using Epi Info 6.01 (CDC, Atlanta, GA, USA), with a significance threshold set at 0.05.

### 2.5 DNA sequencing, alignment and phylogenetic analysis

Positive PCR products obtained from the amplification of mitochondrial 16S rRNA, *ompB, ompA* and *gltA* partial sequences of *Rickettsia* spp. were purified using the GF-1 Ambi Clean kit (Vivantis, Oceanside, CA, USA) following the manufacturer's protocol. The resulting purified DNA amplicons were sequenced in both directions, utilizing the same primers employed in the mitochondrial 16S rRNA *ompA* and *gltA* single PCRs, as well as the secondary PCR from the nested amplifications for the *ompB* partial sequences. Sequencing was conducted using the Big Dye Terminator Cycle Sequencing Kit (Applied Biosystems, Foster City, CA, USA) alongside an ABI3730XL automated DNA sequencer (Macrogen Europe, Amsterdam, The Netherlands). Chromatograms were analyzed using Chromas v.2.01. Raw sequences were obtained from both forward and reverse strands to ensure maximum accuracy. Complementary strands of each sequenced product were manually assembled using DNAMAN software v.5.2.2. (Lynnon Biosoft, Que., Canada). Additionally, overlapping regions were identified following the automated removal of primer sequences. The nucleotide sequences from the mitochondrial 16S rRNA marker, as well as the *ompB, ompA*, and *gltA* genes of *Rickettsia* spp., were utilized to calculate genotype diversity (Gd), nucleotide diversity (Pi) and the average number of nucleotide differences (k) using DnaSP v 5.10. Sequence similarities were determined using the CLUSTAL W v.1.81 method after performing multiple sequence alignments. A BLAST analysis was conducted to evaluate the similarity levels with previously reported sequences in GenBank (http://blast.ncbi.nlm.nih.gov/). Genetic distances among the operational taxonomic units were calculated using the maximum composite likelihood method through DNAMAN software, which were then utilized to construct Maximum Likelihood trees (Tamura and Nei, 1993). A bootstrapping process with 1,000 iterations was employed to assess the statistical support for the internal branches of these trees (Felsenstein and Sinauer, 2008).

## 3 Results

### 3.1 Morphological and molecular identification of ticks and phylogenetic analysis

#### 3.1.1 Efficiency of DNA isolation and distribution of collected ticks

A total of 236 ticks (160 females, 52 males, and 24 nymphs) were collected from a single site in the Jouza district, Beja, Tunisia (Figure 1), comprising 167 ticks sampled from vegetation and 69 ticks removed from a single red fox found at this location. Genomic DNA was extracted from all specimens, followed by PCR amplification targeting the mitochondrial 16S rRNA, which yielded successful amplification in 100% of the samples, confirming the efficiency of the DNA isolation method. Morphological identification, based on the diagnostic keys of (Walker et al. 2005) and (Estrada-Peña et al. 2017), classified nymph ticks (*n* = 24) as belonging to the *Ixodes* genus, and adult ticks into two complexes: *Ix. ricinus* complex (*n* = 94) and *Rh. sanguineus* sensu lato (s.l.) complex *(n* = 78). Molecular identification through sequencing of all *Ixodes* PCR products and BLAST analysis of partial 16S rRNA sequences revealed that, within the *Ix. ricinus* complex, *Ix. inopinatus* (*n* = 89) and Ix. ricinus (*n* = 34) were identified, while all nymphs (*n* = 24) were classified as *Ix. hexagonus* (Table 2). Additionally, for the *Rh. sanguineus* s.l. complex, sequencing of specimens positive for *Rickettsia* spp. and subsequent BLAST analysis identified *Rh. sanguineus* (temperate lineage) (*n* = 24) and *Rh. rutilus* (*n* = 5) (Table 2). To prevent and monitor potential contamination, particularly important for nested PCR protocols, all reactions were conducted in separate areas with unidirectional workflow, and negative controls were systematically included in each PCR run (Supplementary Figure S1A).

**Table 2:** Molecular prevalence results of *Rickettsia* spp. infecting *Ixodes* spp. and *Rhipicephalus sanguineus* sensu lato complex ticks according to several risk factors.

**Factors**	**Number**	**Positive (% ±C.I.^a^)**	***P-*value**
Tick species or complex			0.000^*^
*Ixodes inopinatus*	89	60 (67.4 ± 0.09)	
*Ixodes ricinus*	45	34 (75.6 ± 0.12)	
*Ixodes hexagonus*	24	0 (0)	
*Rhipicephalus sanguineus* sensu lato	78	29 (37.2 ± 0.10)	
Tick gender or developmental stage			0.000^*^
Male	52	34 (65.4 ± 0.12)	
Female	160	89 (55.6 ± 0.07)	
Nymph	24	0 (0)	
Host or environment			0.000^*^
Vegetation	167	101 (60.5 ± 0.07)	
Red fox	69	22 (31.9 ± 0.10)	
Total	236	123 (52.1 ± 0.06)	

^a^C.I., 95% confidence interval.

^*^Statistically significant test.

#### 3.1.2 Genotyping and phylogenetic analysis of selected tick specimens

Partial sequence analysis of the mitochondrial 16S rRNA revealed genotypic variation among the collected tick specimens, with distinct genotypes differing by at least one nucleotide mutation in the mitochondrial 16S rRNA.

##### 3.1.2.1 Genetic diversity and phylogeny of *Ixodes* spp. isolates

**Figure 2:**
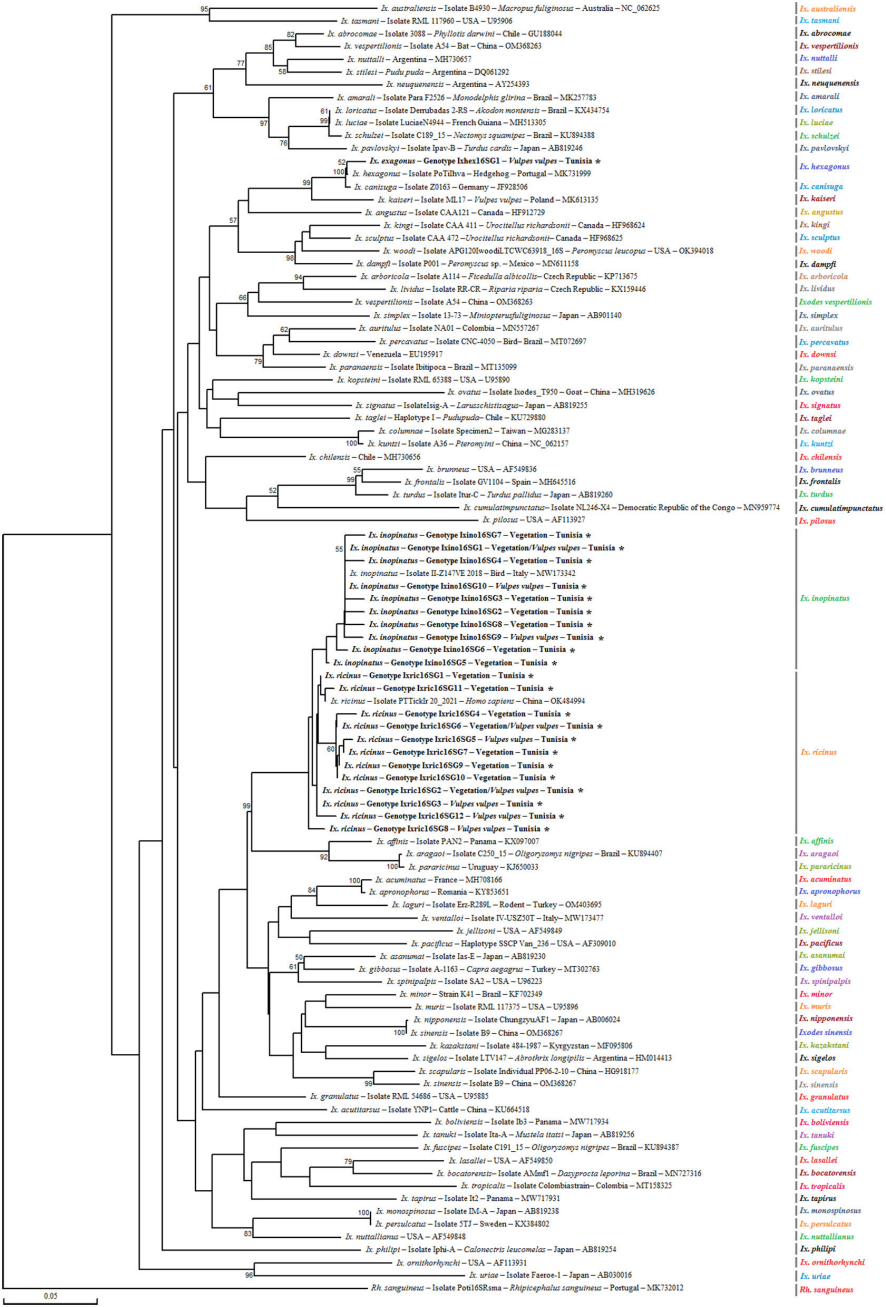
Phylogenetic analysis of partial (320 bp) mitochondrial 16S rRNA sequences obtained from *Ixodes ricinus* complex tick specimens and reference sequences from other *Ixodes* species available in GenBank, using the Neighbor-Joining method. Bootstrap values based on 1,000 iterations are shown at the nodes (only values above 50% are displayed). Information on host, strain, isolate or clone, country of origin, and GenBank accession number is provided. Sequences from the *Ixodes ricinus* complex generated in this study are shown in bold and marked with an asterisk. A partial mitochondrial 16S rRNA sequence from *Rhipicephalus sanguineus* was included as an outgroup.

*Ixodes ricinus* specimens, collected from both vegetation and *V. vulpes* in the Jouza district (Beja, Tunisia), exhibited substantial genetic diversity. A total of 12 distinct genotypes were identified (Ixric16SG1–Ixric16SG12) (Table 3 and Supplementary File 1). Genotypic diversity (Gd) was estimated at 0.539, with a GC content of 51.1%. Nucleotide diversity (Pi) was 0.00224, and the mean number of pairwise nucleotide difference (k) was 0.614 (Table 4). Phylogenetic tree revealed that genotypes such as Ixric16SG2 and Ixric16SG6 were detected in vegetation and *V. vulpes* (Figure 2). Conversely, Ixric16SG1, Ixric16SG4, Ixric16SG7, Ixric16SG9, Ixric16SG10, and Ixric16SG11 were exclusively associated with questing ticks, and Ixric16SG3, Ixric16SG5, Ixric16SG8, Ixric16SG12 to *V. vulpes* (Figure 2). Notably, Ixric16SG1 and Ixric16SG11 clustered with an *Ix. ricinus* isolate from *Homo sapiens* in China (GenBank accession number: OK484994), while Ixric16SG12 was detected solely in *V. vulpes*, clustering within the principal *Ix. ricinus* lineage (Figure 2).

**Table 3:** Designation and information on the origins and genotypes of Tunisian isolates of *Rickettsia* spp. isolated from *Ix. ricinus* ticks collected from vegetation and a red fox (*Vulpes vulpes*) in Jouza district (Beja governorate, Tunisia).

**Sample**	**Morp. Id**.	**Host or environment**	**BLAST^a^ (GenBank^b^, Genotype)**	**BLAST**^**c**^ **(GenBank**^**b**^**, Genotype)**		
				***ompB***	***ompA***	***gltA***
Ixric4	*Ix. ricinus* complex	Vegetation	99.6% *Ix. ricinus* (PV018108, Ixric16SG1)	100% *R. monacensis* (PV029551, RmonompBG1)	99.8% *R. monacensis* (PV032631, RmonompAG1)	100% *R. monacensis* (PV029620, RmongltAG1)
Ixric8	*Ix. ricinus* complex	Vegetation	100% *Ix. ricinus* (PV018109, Ixric16SG2)	100% *R. monacensis* (PV029552, RmonompBG1)	99.8% *R. monacensis* (PV032632, RmonompAG1)	100% *R. monacensis* (PV029621, RmongltAG1)
Ixric133	*Ix. ricinus* complex	*Vulpes vulpes*	99.6% *Ix. ricinus* (PV018110, Ixric16SG3)	100% *R. monacensis* (PV029553, RmonompBG1)	99.8% *R. monacensis* (PV032633, RmonompAG1)	100% *R. monacensis* (PV029622, RmongltAG1)
Ixric20	*Ix. ricinus* complex	Vegetation	100% *Ix. ricinus* (PV018111, Ixric16SG4)	100% *R. monacensis* (PV029554, RmonompBG1)	99.8% *R. monacensis* (PV032634, RmonompAG1)	–
Ixic169	*Ix. ricinus* complex	*Vulpes vulpes*	100% *Ix. ricinus* (PV018112, Ixric16SG5)	100% *R. monacensis* (PV029555, RmonompBG1)	99.8% *R. monacensis* (PV032635, RmonompAG1)	–
Ixric170	*Ix. ricinus* complex	*Vulpes vulpes*	100% *Ix. ricinus* (PV018113, Ixric16SG6)	100% *R. monacensis* (PV029556, RmonompBG1)	99.8% *R. monacensis* (PV032636, RmonompAG1)	–
Ixric92	*Ix. ricinus* complex	Vegetation	100% *Ix. ricinus* (PV018114, Ixric16SG7)	100% *R. helvetica* (PV029600, RhelompBG1)	–	99.7% *R. helvetica* (PV029638, RhelgltAG1)
Ixric123	*Ix. ricinus* complex	*Vulpes vulpes*	99.6% *Ix. ricinus* (PV018115, Ixric16SG8)	100% *R. monacensis* (PV029557, RmonompBG1)	–	100% *R. monacensis* (PV029623, RmongltAG1)
Ixric11	*Ix. ricinus* complex	Vegetation	100% *Ix. ricinus* (PV018116, Ixric16SG9)	100% *R. helvetica* (PV029601, RhelompBG1)	–	–
Ixric13	*Ix. ricinus* complex	Vegetation	100% *Ix. ricinus* (PV018117, Ixric16SG2)	100% *R. helvetica* (PV029602, RhelompBG1)	–	–
Ixric22	*Ix. ricinus* complex	Vegetation	100% *Ix. ricinus* (PV018118, Ixric16SG10)	100% *R. monacensis* (PV029558, RmonompBG1)	–	–
Ixric23	*Ix. ricinus* complex	Vegetation	100% *Ix. ricinus* (PV018119, Ixric16SG6)	100% *R. monacensis* (PV029559, RmonompBG1)	–	–
Ixric42	*Ix. ricinus* complex	Vegetation	100% *Ix. ricinus* (PV018120, Ixric16SG2)	100% *R. monacensis* (PV029560, RmonompBG1)	–	–
Ixric50	*Ix. ricinus* complex	Vegetation	100% *Ix. ricinus* (PV018121, Ixric16SG10)	100% *R. monacensis* (PV029561, RmonompBG1)	–	–
Ixric74	*Ix. ricinus* complex	Vegetation	100% *Ix. ricinus* (PV018122, Ixric16SG6)	100% *R. monacensis* (PV029562, RmonompBG1)	–	–
Ixric76	*Ix. ricinus* complex	Vegetation	100% *Ix. ricinus* (PV018123, Ixric16SG2)	99.7% *R. monacensis* (PV029563, RmonompBG2)	–	–
Ixric102	*Ix. ricinus* complex	Vegetation	100% *Ix. ricinus* (PV018124, Ixric16SG2)	100% *R. monacensis* (PV029564, RmonompBG1)	–	–
Ixric104	*Ix. ricinus* complex	Vegetation	100% *Ix. ricinus* (PV018125, Ixric16SG6)	100% *R. helvetica* (PV029603, RhelompBG1)	–	–
Ixric121	*Ix. ricinus* complex	*Vulpes vulpes*	100% *Ix. ricinus* (PV018126, Ixric16SG6)	100% *R. helvetica* (PV029604, RhelompBG1)	–	–
Ixric141	*Ix. ricinus* complex	*Vulpes vulpes*	100% *Ix. ricinus* (PV018127, Ixric16SG6)	100% *R. helvetica* (PV029605, RhelompBG1)	–	–
Ixric172	*Ix. ricinus* complex	*Vulpes vulpes*	100% *Ix. ricinus* (PV018128, Ixric16SG5)	–	99.8% *R. monacensis* (PV032637, RmonompAG1)	–
Ixric37	*Ix. ricinus* complex	Vegetation	100% *Ix. ricinus* (PV018129, Ixric16SG2)	–	99.8% *R. monacensis* (PV032638, RmonompAG1)	–
Ixric39	*Ix. ricinus* complex	Vegetation	99.6% *Ix. ricinus* (PV018130, Ixric16SG11)	–	99.8% *R. monacensis* (PV032639, RmonompAG1)	–
Ixric33	*Ix. ricinus* complex	Vegetation	100% *Ix. ricinus* (PV018131, Ixric16SG2)	–	–	100% *R. monacensis* (PV029624, RmongltAG1)
Ixric34	*Ix. ricinus* complex	Vegetation	100% *Ix. ricinus* (PV018132, Ixric16SG6)	–	–	100% *R. monacensis* (PV029625, RmongltAG1)
Ixric75	*Ix. ricinus* complex	Vegetation	100% *Ix. ricinus* (PV018133, Ixric16SG6)	–	–	100% *R. monacensis* (PV029626, RmongltAG1)
Ixric87	*Ix. ricinus* complex	Vegetation	100% *Ix. ricinus* (PV018134, Ixric16SG2)	–	–	100% *R. helvetica* (PV029639, RhelgltAG2)
Ixric96	*Ix. ricinus* complex	Vegetation	100% *Ix. ricinus* (PV018135, Ixric16SG2)	–	–	100% *R. helvetica* (PV029640, RhelgltAG2)
Ixric98	*Ix. ricinus* complex	Vegetation	100% *Ix. ricinus* (PV018136, Ixric16SG6)	–	–	100% *R. monacensis* (PV029627, RmongltAG1)
Ixric166	*Ix. ricinus* complex	*Vulpes vulpes*	100% *Ix. ricinus* (PV018137, Ixric16SG6)	–	–	100% *R. helvetica* (PV029641, RhelgltAG2)

Morp. Id., morphologically identified tick species; ^a^BLAST analysis for mitochondrial 16S rRNA partial sequence of ticks; ^b^GenBank accession number; ^c^BLAST analysis for ompB, *ompA* and *gltA* partial sequences of *Rickettsia* spp.; Ix. ricinus complex, *Ixodes* ricinus complex composed of*Ixodes* ricinus and*Ixodes* inopinatus; –, not sequenced.

**Table 4:** Genetic diversity found within mitochondrial 16S rRNA partial sequences isolated from selected ticks and *ompB, ompA* and *gltA* partial sequences isolated from *Rickettsia* spp. infecting ticks.

**Gene**	**Tick or *Rickettsia* species**	**Size (pb)^a^**	**N**	**VS^b^**	**GC%^c^**	**G**	**Gd^d^**	**Pi^e^**	**K^f^**
16S rRNA	*Ix. ricinus*	274–276	45	8 (2)	51.1	12	0.539 (0.314)	0.00224 (0.00182)	0.614 (0.501)
	*Ix. inopinatus*	275–277	59	8 (2)	51.6	10	0.255 (0.268)	0.00095 (0.00095)	0.271 (0.272)
	*Ix. hexagonus*	279	24	0	54.9	1	0	0	0
	*Rh. sanguineus* s.s.	272	23	3	50.0	4	0.383	0.00181	0.490
	*Rh. rutilus*	274	5	0	51.1	1	0	0	0
*ompB*	*R. monacensis*	382	49	2	51.6	2	0.080	0.00021	0.080
	*R. helvetica*	382	12	0	50.5	1	0	0	0
	*R. massiliae*	382	8	5	50.6	2	0.429	0.00561	2.143
*ompA*	*R. monacensis*	487	25	0	55.8	1	0	0	0
	*R. massiliae*	490	8	0	54.0	1	0	0	0
*gltA*	*R. monacensis*	341	18	0	48.1	1	0	0	0
	*R. helvetica*	341	9	1	48.4	2	0.222	0.00065	0.222
	*R. massiliae*	341	7	0	49.0	1	0	0	0

N, number of analyzed sequences; VS, Number of variable sites; %GC, percentage in GC; G, number of genotypes; Gd, genotypic diversity; Pi, nucleotide diversity, k, average number of nucleotide differences.

^a^Total number of sites (excluding and including sites with gaps).

^b^The first number is the number of mutations excluding sites with gaps and the second number in brackets is the total number of InDel sites.

^c^Cytosine and Guanine rate (%) according to the first genotype.

^d^The first value is the genotype diversity excluding sites with gaps and the second value in brackets is the InDel genotype diversity.

^e^The first value is the nucleotide diversity excluding sites with gaps and the second value in brackets is the InDel nucleotide diversity.

^f^The first value is the average number of nucleotide differences excluding sites with gaps and the second value in brackets is the InDel average number of nucleotide differences.

On the other hand, for *Ix. inopinatus*, 10 genotypes (Ixino16SG1–Ixino16SG10) were identified (Table 5 and Supplementary File 2). Genotypic diversity (Gd) was 0.255, with a GC content of 51.6%. Nucleotide diversity (Pi) was 0.0095, with a mean pairwise nucleotide difference (k) of 0.271 (Table 4). Phylogenetic analysis confirmed that all identified genotypes belonged to the *Ix. inopinatus* cluster (Figure 2). With the exception of the two genotypes Ixino16SG5 and Ixino16SG6, which clustered closely, all other genotypes are part of a relatively homogeneous sub-cluster that is phylogenetically close to the *Ix. inopinatus* isolate found in a bird in Italy (GenBank accession number: MW173342) (Figure 2). Furthermore, genetic diversity analysis of *Ix. hexagonus* using DnaSP v5.10.01 identified a single genotype (Ixhex16SG1) in all 24 nymphs collected from *V. vulpes* (Supplementary File 3). This genotype exhibited a GC content of 54.9% and shared 98.9% sequence similarity with the PoTiIhva isolate from a Portuguese hedgehog (GenBank accession number: MK731999) (Table 4 and Figure 2).

**Table 5:** Designation and information on the origins and genotypes of Tunisian isolates of *Rickettsia* spp. isolated from *Ix. inopinatus* ticks collected from vegetation and a red fox *(Vulpes vulpes*) in Jouza district (Beja governorate, Tunisia).

**Sample**	**Morp. Id**.	**Host or environment**	**BLAST^a^ (GenBank^b^, Genotype)**	**BLAST**^**c**^ **(GenBank**^**b**^**, Genotype)**		
				***ompB***	***ompA***	***gltA***
Ixinp24	*Ix. ricinus* complex	Vegetation	99.6% *Ix. inopinatus* (PV018153, Ixino16SG1)	100% *R. monacensis* (PV029565, RmonompBG1)	99.8% *R. monacensis* (PV032640, RmonompAG1)	100% *R. monacensis* (PV029628, RmongltAG1)
Ixinp25	*Ix. ricinus* complex	Vegetation	99.6% *Ix. inopinatus* (PV018154, Ixino16SG1)	100% *R. monacensis* (PV029566, RmonompBG1)	99.8% *R. monacensis* (PV032641, RmonompAG1)	100% *R. monacensis* (PV029629, RmongltAG1)
Ixinp35	*Ix. ricinus* complex	Vegetation	99.6% *Ix. inopinatus* (PV018155, Ixino16SG1)	100% *R. monacensis* (PV029567, RmonompBG1)	99.8% *R. monacensis* (PV032642, RmonompAG1)	100% *R. monacensis* (PV029630, RmongltAG1)
Ixinp43	*Ix. ricinus* complex	Vegetation	99.6% *Ix. inopinatus* (PV018156, Ixino16SG1)	100% *R. monacensis* (PV029568, RmonompBG1)	99.8% *R. monacensis* (PV032643, RmonompAG1)	100% *R. monacensis* (PV029631, RmongltAG1)
Ixinp124	*Ix. ricinus* complex	*Vulpes vulpes*	99.6% *Ix. inopinatus* (PV018157, Ixino16SG1)	100% *R. monacensis* (PV029569, RmonompBG1)	99.8% *R. monacensis* (PV032644, RmonompAG1)	100% *R. monacensis* (PV029632, RmongltAG1)
Ixinp158	*Ix. ricinus* complex	*Vulpes vulpes*	99.6% *Ix. inopinatus* (PV018158, Ixino16SG1)	100% *R. monacensis* (PV029570, RmonompBG1)	99.8% *R. monacensis* (PV032645, RmonompAG1)	–
Ixinp171	*Ix. ricinus* complex	*Vulpes vulpes*	99.6% *Ix. inopinatus* (PV018159, Ixino16SG1)	100% *R. monacensis* (PV029571, RmonompBG1)	99.8% *R. monacensis* (PV032646, RmonompAG1)	–
Ixinp6	*Ix. ricinus* complex	Vegetation	99.6% *Ix. inopinatus* (PV018160, Ixino16SG1)	100% *R. helvetica* (PV029606, RhelompBG1)	–	100% *R. helvetica* (PV029642, RhelgltAG2)
Ixinp85	*Ix. ricinus* complex	Vegetation	99.6% *Ix. inopinatus* (PV018161, Ixino16SG1)	100% *R. monacensis* (PV029572, RmonompBG1)	–	100% *R. helvetica* (PV029643, RhelgltAG2)
Ixinp131	*Ix. ricinus* complex	*Vulpes vulpes*	99.6% *Ix. inopinatus* (PV018162, Ixino16SG1)	100% *R. monacensis* (PV029573, RmonompBG1)	–	100% *R. monacensis* (PV029633, RmongltAG1)
Ixinp155	*Ix. ricinus* complex	*Vulpes vulpes*	99.6% *Ix. inopinatus* (PV018163, Ixino16SG1)	–	99.8% *R. monacensis* (PV032647, RmonompAG1)	100% *R. monacensis* (PV029634, RmongltAG1)
Ixinp174	*Ix. ricinus* complex	*Vulpes vulpes*	99.6% *Ix. inopinatus* (PV018164, Ixino16SG1)	–	99.8% *R. monacensis* (PV032648, RmonompAG1)	100% *R. monacensis* (PV029635, RmongltAG1)
Ixinp3	*Ix. ricinus* complex	Vegetation	99.6% *Ix. inopinatus* (PV018165, Ixino16SG2)	100% *R. monacensis* (PV029574, RmonompBG1)	–	–
Ixinp7	*Ix. ricinus* complex	Vegetation	99.6% *Ix. inopinatus* (PV018166, Ixino16SG1)	100% *R. monacensis* (PV029575, RmonompBG1)	–	–
Ixinp10	*Ix. ricinus* complex	Vegetation	99.6% *Ix. inopinatus* (PV018167, Ixino16SG1)	100% *R. helvetica* (PV029607, RhelompBG1)	–	–
Ixinp12	*Ix. ricinus* complex	Vegetation	99.6% *Ix. inopinatus* (PV018168, Ixino16SG1)	100% *R. monacensis* (PV029576, RmonompBG1)	–	–
Ixinp18	*Ix. ricinus* complex	Vegetation	99.6% *Ix. inopinatus* (PV018169, Ixino16SG1)	100% *R. monacensis* (PV029577, RmonompBG1)	–	–
Ixinp27	*Ix. ricinus* complex	Vegetation	99.6% *Ix. inopinatus* (PV018170, Ixino16SG1)	100% *R. monacensis* (PV029578, RmonompBG1)	–	–
Ixinp29	*Ix. ricinus* complex	Vegetation	99.6% *Ix. inopinatus* (PV018171, Ixino16SG1)	99.7% *R. monacensis* (PV029579, RmonompBG2)	–	–
Ixinp31	*Ix. ricinus* complex	Vegetation	99.6% *Ix. inopinatus* (PV018172, Ixino16SG1)	100% *R. monacensis* (PV029580, RmonompBG1)	–	–
Ixinp36	*Ix. ricinus* complex	Vegetation	99.6% *Ix. inopinatus* (PV018173, Ixino16SG1)	100% *R. monacensis* (PV029581, RmonompBG1)	–	–
Ixinp38	*Ix. ricinus* complex	Vegetation	99.6% *Ix. inopinatus* (PV018174, Ixino16SG1)	100% *R. helvetica* (PV029608, RhelompBG1)	–	–
Ixinp45	*Ix. ricinus* complex	Vegetation	99.6% *Ix. inopinatus* (PV018175, Ixino16SG3)	100% *R. helvetica* (PV029609, RhelompBG1)	–	–
Ixinp48	*Ix. ricinus* complex	Vegetation	99.6% *Ix. inopinatus* (PV018176, Ixino16SG1)	100% *R. monacensis* (PV029582, RmonompBG1)	–	–
Ixinp51	*Ix. ricinus* complex	Vegetation	99.6% *Ix. inopinatus* (PV018177, Ixino16SG1)	100% *R. monacensis* (PV029583, RmonompBG1)	–	–
Ixinp70	*Ix. ricinus* complex	Vegetation	99.6% *Ix. inopinatus* (PV018178, Ixino16SG1)	100% *R. monacensis* (PV029584, RmonompBG1)	–	–
Ixinp71	*Ix. ricinus* complex	Vegetation	99.6% *Ix. inopinatus* (PV018179, Ixino16SG1)	100% *R. helvetica* (PV029610, RhelompBG1)	–	–
Ixinp72	*Ix. ricinus* complex	Vegetation	99.6% *Ix. inopinatus* (PV018180, Ixino16SG1)	100% *R. monacensis* (PV029585, RmonompBG1)	–	–
Ixinp77	*Ix. ricinus* complex	Vegetation	99.6% *Ix. inopinatus* (PV018181, Ixino16SG1)	100% *R. monacensis* (PV029586, RmonompBG1)	–	–
Ixinp78	*Ix. ricinus* complex	Vegetation	99.6% *Ix. inopinatus* (PV018182, Ixino16SG4)	100% *R. monacensis* (PV029587, RmonompBG1)	–	–
Ixinp79	*Ix. ricinus* complex	Vegetation	99.6% *Ix. inopinatus* (PV018183, Ixino16SG1)	100% *R. monacensis* (PV029588, RmonompBG1)	–	–
Ixinp82	*Ix. ricinus* complex	Vegetation	99.6% *Ix. inopinatus* (PV018184, Ixino16SG1)	100% *R. monacensis* (PV029589, RmonompBG1)	–	–
Ixinp83	*Ix. ricinus* complex	Vegetation	99.6% *Ix. inopinatus* (PV018185, Ixino16SG1)	100% *R. monacensis* (PV029590, RmonompBG1)	–	–
Ixinp84	*Ix. ricinus* complex	Vegetation	99.6% *Ix. inopinatus* (PV018186, Ixino16SG1)	100% *R. monacensis* (PV029591, RmonompBG1)	–	–
Ixinp86	*Ix. ricinus* complex	Vegetation	99.6% *Ix. inopinatus* (PV018187, Ixino16SG1)	100% *R. monacensis* (PV029592, RmonompBG1)	–	–
Ixinp91	*Ix. ricinus* complex	Vegetation	99.6% *Ix. inopinatus* (PV018188, Ixino16SG1)	100% *R. monacensis* (PV029593, RmonompBG1)	–	–
Ixinp93	*Ix. ricinus* complex	Vegetation	99.6% *Ix. inopinatus* (PV018189, Ixino16SG1)	100% *R. monacensis* (PV029594, RmonompBG1)	–	–
Ixinp95	*Ix. ricinus* complex	Vegetation	99.6% *Ix. inopinatus* (PV018190, Ixino16SG1)	100% *R. monacensis* (PV029595, RmonompBG1)	–	–
Ixinp101	*Ix. ricinus* complex	Vegetation	99.6% *Ix. inopinatus* (PV018191, Ixino16SG1)	100% *R. monacensis* (PV029596, RmonompBG1)	–	–
Ixinp162	*Ix. ricinus* complex	*Vulpes vulpes*	99.6% *Ix. inopinatus* (PV018192, Ixino16SG1)	100% *R. helvetica* (PV029611, RhelompBG1)	–	–
Ixinp165	*Ix. ricinus* complex	*Vulpes vulpes*	99.6% *Ix. inopinatus* (PV018193, Ixino16SG1)	100% *R. monacensis* (PV029597, RmonompBG1)	–	–
Ixinp167	*Ix. ricinus* complex	*Vulpes vulpes*	99.6% *Ix. inopinatus* (PV018194, Ixino16SG1)	100% *R. monacensis* (PV029598, RmonompBG1)	–	–
Ixinp175	*Ix. ricinus* complex	*Vulpes vulpes*	99.6% *Ix. inopinatus* (PV018195, Ixino16SG1)	100% *R. monacensis* (PV029599, RmonompBG1)	–	–
Ixinp49	*Ix. ricinus* complex	Vegetation	100% *Ix. inopinatus* (PV018196, Ixino16SG5)	–	99.8% *R. monacensis* (PV032649, RmonompAG1)	–
Ixinp67	*Ix. ricinus* complex	Vegetation	99.6% *Ix. inopinatus* (PV018197, Ixino16SG1)	–	99.8% *R. monacensis* (PV032650, RmonompAG1)	–
Ixinp89	*Ix. ricinus* complex	Vegetation	99.6% *Ix. inopinatus* (PV018198, Ixino16SG1)	–	99.8% *R. monacensis* (PV032651, RmonompAG1)	–
Ixinp94	*Ix. ricinus* complex	Vegetation	99.6% *Ix. inopinatus* (PV018199, Ixino16SG6)	–	99.8% *R. monacensis* (PV032652, RmonompAG1)	–
Ixinp106	*Ix. ricinus* complex	Vegetation	99.6% *Ix. inopinatus* (PV018200, Ixino16SG1)	–	99.8% *R. monacensis* (PV032653, RmonompAG1)	–
Ixinp107	*Ix. ricinus* complex	*Vulpes vulpes*	99.6% *Ix. inopinatus* (PV018201, Ixino16SG1)	–	99.8% *R. monacensis* (PV032654, RmonompAG1)	–
Ixinp161	*Ix. ricinus* complex	*Vulpes vulpes*	99.6% *Ix. inopinatus* (PV018202, Ixino16SG1)	–	99.8% *R. monacensis* (PV032655, RmonompAG1)	–
Ixinp5	*Ix. ricinus* complex	Vegetation	99.6% *Ix. inopinatus* (PV018203, Ixino16SG1)	–	–	100% *R. helvetica* (PV029644, RhelgltAG2)
Ixinp46	*Ix. ricinus* complex	Vegetation	99.6% *Ix. inopinatus* (PV018204, Ixino16SG1)	–	–	100% *R. helvetica* (PV029645, RhelgltAG2)
Ixinp52	*Ix. ricinus* complex	Vegetation	99.6% *Ix. inopinatus* (PV018205, Ixino16SG1)	–	–	100% *R. helvetica* (PV029646, RhelgltAG2)
Ixinp99	*Ix. ricinus* complex	Vegetation	99.6% *Ix. inopinatus* (PV018206, Ixino16SG1)	–	–	100% *R. monacensis* (PV029636, RmongltAG1)
Ixinp129	*Ix. ricinus* complex	*Vulpes vulpes*	99.6% *Ix. inopinatus* (PV018207, Ixino16SG1)	–	–	100% *R. monacensis* (PV029637, RmongltAG1)

Morp. Id., morphologically identified tick species; ^a^BLAST analysis for mitochondrial 16S rRNA partial sequence of ticks; ^b^GenBank accession number; ^c^BLAST analysis for ompB, *ompA* and *gltA* partial sequences of *Rickettsia* spp.; Ix. ricinus complex, *Ixodes* ricinus complex composed of*Ixodes* ricinus and*Ixodes* inopinatus; –, not sequenced.

##### 3.1.2.2 Genetic diversity and phylogeny within the *Rh sanguineus* s.l. complex

**Figure 3:**
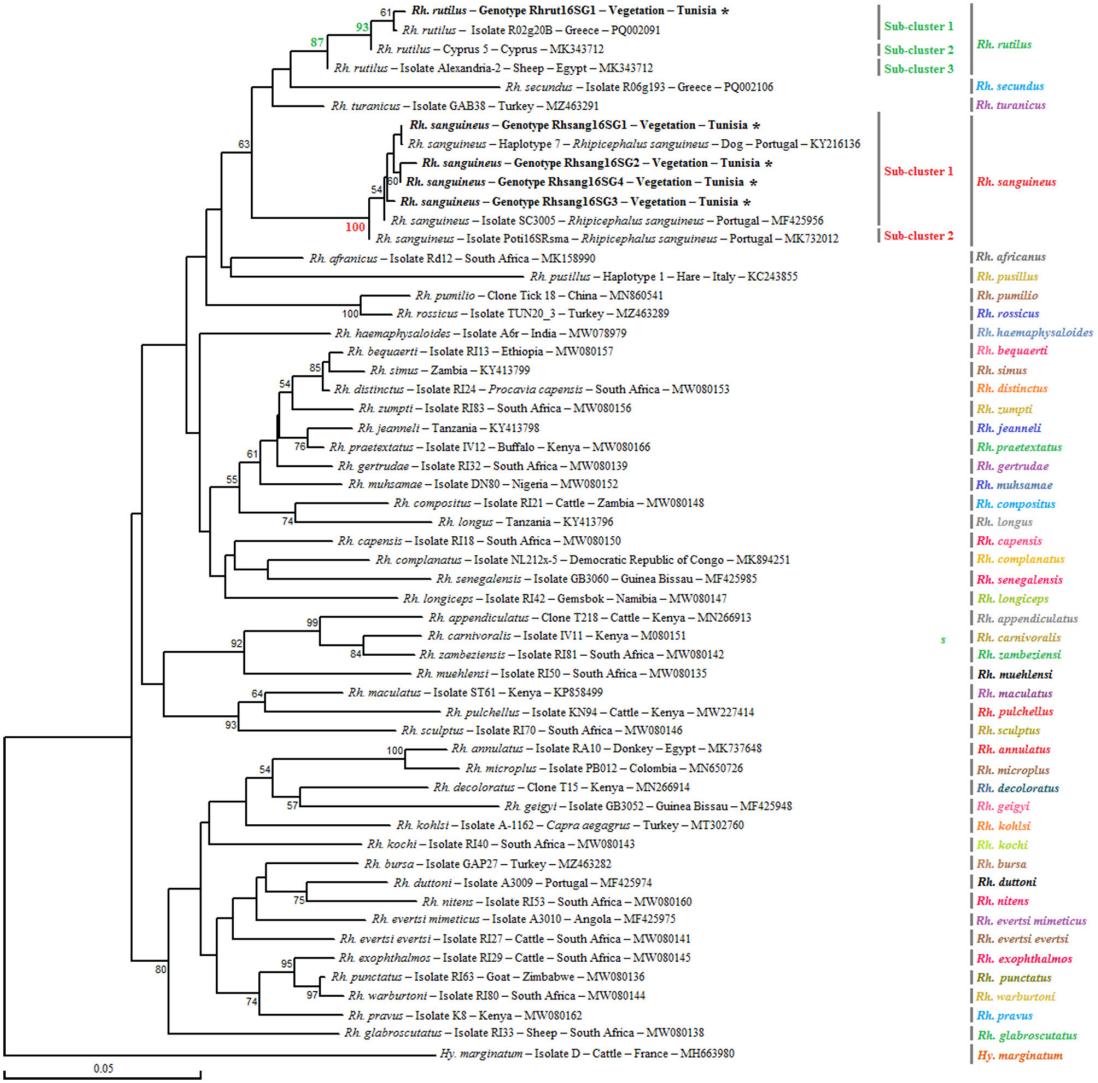
Phylogenetic analysis of partial (320 bp) mitochondrial 16S rRNA sequences obtained from tick specimens identified as *Rhipicephalus sanguineus* sensu lato and reference sequences from other *Rhipicephalus* species available in GenBank, using the Neighbor-Joining method. Bootstrap values based on 1,000 iterations are shown at the nodes (only values above 50% are displayed). Information on host, strain, isolate or clone, country of origin, and GenBank accession number is provided. Sequences of *Rh. sanguineus* s.l. generated in this study are shown in bold and marked with an asterisk. A partial mitochondrial 16S rRNA sequence from *Hyalomma marginatum* was included as an outgroup.

Analysis of the partial 16S rRNA sequence of *Rh. sanguineus* s.l. specimens collected from vegetation in Jouza identified four genotypes, designated as Rhsan16SG1-Rhsan16SG4 (Table 6 and Supplementary File 4). Genotypic diversity (Gd) was 0.421, with a GC content of 50%. Nucleotide diversity (Pi) was 0.0089, and k was 0.512 (Table 4). Interestingly, a single genotype (Rhrut16SG1) was identified in five *Rh. rutilus* specimens (Tables 4, 6 and Supplementary File 4). Phylogenetic analysis revealed that the group of *Rh. sanguineus* s.l. comprises four tick species within the *Rhipicephalus* genus, namely *Rh. sanguineus* sensu strito, *Rh. rutilus, Rh. secundus*, and *Rh. turanicus* (Figure 3). *Rh. sanguineus* s.s. cluster was composed of two sub-clusters supported by bootstrap values of 100% (Figure 3). All our genotypes of *Rh. sanguineus* s.s. were classified within sub-cluster 1, along with Haplotype 7 of *Rh. sanguineus* s.s. infecting a dog in Portugal (GenBank accession number: KY216136) (Figure 3). *Rh. rutilus* cluster was composed of three sub-clusters, with a bootstrap value of 93% between sub-cluster 1 and sub-cluster 2, and 87% between sub-cluster 2 and sub-cluster 3 (Figure 3). The genotype identified in this study of *Rh. rutilus* was present in sub-cluster 1, along with the isolate R02G20B of *Rh. rutilus* from Greece (GenBank accession number: PQ002091) (Figure 3).

**Table 6:** Designation and information on the origins and genotypes of Tunisian isolates of *Rickettsia* spp. isolated from *Rh. rutilus* and *Rh. sanguineus* ticks collected from vegetation in Jouza district (Beja governorate, Tunisia).

**Sample**	**Morp. Id**.	**Host or environment**	**BLAST^a^ (GenBank^b^, Genotype)**	**BLAST**^**c**^ **(GenBank**^**b**^**, Genotype)**		
				***ompB***	***ompA***	***gltA***
Rhrut2	*Rh. sanguineus* s.l.	Vegetation	100% *Rh. rutilus* (PV018265, Rhrut16SG1)	100% *R. massiliae* (PV029612, RmasompBG1)	100% *R. massiliae* (PV032656, RmasompAG1)	100% *R. massiliae* (PV029647, RmasgltAG1)
Rhrut27	*Rh. sanguineus* s.l.	Vegetation	100% *Rh. rutilus* (PV018266, Rhrut16SG1)	100% *R. massiliae* (PV029613, RmasompBG2)	100% *R. massiliae* (PV032657, RmasompAG1)	–
Rhrut15	*Rh. sanguineus* s.l.	Vegetation	100% *Rh. rutilus* (PV018267, Rhrut16SG1)	100% *R. massiliae* (PV029614, RmasompBG1)	–	–
Rhrut4	*Rh. sanguineus* s.l.	Vegetation	100% *Rh. rutilus* (PV018268, Rhrut16SG1)	–	–	100% *R. massiliae* (PV029648, RmasgltAG1)
Rhsan13	*Rh. sanguineus* s.l.	Vegetation	100% *Rh. sanguineus* (PV018270, Rhsan16SG1)	100% *R. massiliae* (PV029615, RmasompBG1)	100% *R. massiliae* (PV032658, RmasompAG1)	100% *R. massiliae* (PV029649, RmasgltAG1)
Rhsan9	*Rh. sanguineus* s.l.	Vegetation	99.2% *Rh. sanguineus* (PV018271, Rhsan16SG2)	100% *R. massiliae* (PV029616, RmasompBG1)	100% *R. massiliae* (PV032659, RmasompAG1)	–
Rhsan28	*Rh. sanguineus* s.l.	Vegetation	100% *Rh. sanguineus* (PV018272, Rhsan16SG1)	100% *R. massiliae* (PV029617, RmasompBG2)	100% *R. massiliae* (PV032660, RmasompAG1)	–
Rhsan20	*Rh. sanguineus* s.l.	Vegetation	100% *Rh. sanguineus* (PV018273, Rhsan16SG1)	100% *R. massiliae* (PV029618, RmasompBG1)	–	100% *R. massiliae* (PV029650, RmasgltAG1)
Rhsan34	*Rh. sanguineus* s.l.	Vegetation	100% *Rh. sanguineus* (PV018274, Rhsan16SG1)	100% *R. massiliae* (PV029619, RmasompBG1)	–	–
Rhsan8	*Rh. sanguineus* s.l.	Vegetation	100% *Rh. sanguineus* (PV018275, Rhsan16SG1)	–	100% *R. massiliae* (PV032661, RmasompAG1)	–
Rhsan12	*Rh. sanguineus* s.l.	Vegetation	100% *Rh. sanguineus* (PV018276, Rhsan16SG1)	–	100% *R. massiliae* (PV032662, RmasompAG1)	–
Rhsan16	*Rh. sanguineus* s.l.	Vegetation	–	–	100% *R. massiliae* (PV032663, RmasompAG1)	–
Rhsan3	*Rh. sanguineus* s.l.	Vegetation	100% *Rh. sanguineus* (PV018277, Rhsan16SG1)	–	–	100% *R. massiliae* (PV029651, RmasgltAG1)
Rhsan24	*Rh. sanguineus* s.l.	Vegetation	–	–	–	100% *R. massiliae* (PV029652, RmasgltAG1)
Rhsan51	*Rh. sanguineus* s.l.	Vegetation	–	–	–	100% *R. massiliae* (PV029653, RmasgltAG1)

Morp. Id., morphologically identified tick species; ^a^BLAST analysis for mitochondrial 16S rRNA partial sequence of ticks; ^b^GenBank accession number; ^c^BLAST analysis for ompB, *ompA* and *gltA* partial sequences of *Rickettsia* spp.; Rh. sanguineus s.l., *Rhipicephalus* sanguineus sensu lato; –, not sequenced.

### 3.2 Molecular prevalence of *Rickettsia* spp.

PCR amplification targeting *ompB* revealed an overall *Rickettsia* spp. infection rate of 52.1% (123/236) (Table 2). Infection rates were comparable between the two tick species of the *Ix. ricinus* complex, with *Ix. inopinatus* at 67.4% and *Ix. ricinus* at 75.6% (Table 2). In contrast, the *Rh. sanguineus* s.l. complex exhibited a lower infection rate of 37.2% (Table 2). Notably, all *Ix. hexagonus* samples tested negative for *Rickettsia* sp. (0%) (Table 2). These differences were statistically significant (*p* < 0.001; Table 2). Additionally, infection prevalence varied by gender and developmental stage, with rates of 65.4% in males, 55.6% in females, and 0% in nymphs, showing a statistically significant difference (*p* < 0.001; Table 2). Furthermore, distinct infection rates were observed based on collection source, with statistically significant differences (*p* < 0.001; Table 2). Infection rates included 60.5% (101 out of 167) in vegetation, 31.9% (22 out of 69) in the red fox host (Table 2).

### 3.3 *Rickettsia* species identification

To identify and genetically characterize the detected *Rickettsia* species, at least one of the three targeted genes (*ompB, ompA*, and *gltA*) was partially sequenced for the 123 *Rickettsia*-positive tick samples selected for analysis (*Ix. inopinatus, n* = 60; *Ix. ricinus, n* = 30; *Rh. sanguineus* s.l., *n* = 29) (Table 7). All 123 partial sequences were successfully obtained and deposited in GenBank under the accession numbers PV029551–PV029619 for *ompB*, PV032631–PV032663 for *ompA*, and PV029620–PV029653 for *gltA*. Three *Rickettsia* species were identified in ticks positive for *Rickettsia* spp. selected for genetic analysis namely *R. monacensis, R. helvetica*, and *R. massiliae* (Table 7). Furthermore, based on the analysis of the three genes, coinfection by *R. monacensis* and *R. helvetica* was reported in one specimen of *Ix. inopinatus* tick (Ixinp85) (Table 7). Furthermore, to validate the specificity and reliability of the PCR assays used for *Rickettsia* detection, amplification products of the *ompB* (425 bp), *ompA* (532 bp), and *gltA* (381 bp) genes were analyzed by electrophoresis on 1.5% agarose gels stained with ethidium bromide. Each PCR run included both positive controls (DNA from confirmed *Rickettsia* spp.) and negative controls (nuclease-free water). Clear, specific bands of the expected sizes were observed in positive controls and sample lanes, while no amplification was detected in the negative controls, confirming the absence of contamination and the efficiency of the assays (Supplementary Figures S1B–S1D).

**Table 7:** *Rickettsia* species identified by sequencing of partial *ompB, ompA* and *gltA* gene sequences infecting *Ixodes* and *Rhipicephalus sanguineus* sensu lato ticks.

**Tick species**	***ompB* PCR positive/ sequencing**	***omp*A PCR positive/sequencing**	***gltA* PCR positive/sequencing**	***Rickettsia* spp**.
*Ixodes ricinus*	14	9	8	*R. monacensis*
	6	0	4	*R. helvetica*
	0	0	0	*R. massiliae*
*Ixodes inopinatus*	35	16	10	*R. monacensis*
	6	0	5	*R. helvetica*
	0	0	0	*R. massiliae*
*Ixodes hexagonus*	0	0	0	*R. monacensis*
	0	0	0	*R. helvetica*
	0	0	0	*R. massiliae*
*Rhipicephalus sanguineus* sensu lato	0	0	0	*R. monacensis*
	0	0	0	*R. helvetica*
	8	8	7	*R. massiliae*
Total	69	33	34	*Rickettsia* spp.

### 3.4 Genotyping and phylogenetic analysis

For the three genes analyzed, we identified distinct genotypes, each defined by at least one nucleotide difference.

#### 3.4.1 Rickettsia spp. *ompB* partial sequences

The sequencing of *ompB* partial sequence (382 bp) was performed on 69 tick samples belonging to *Ix. ricinus* (*n* = 20), *Ix. inopinatus* (*n* = 41), and *Rh. sanguineus* s.l. (*n* = 8) (Table 7). The BLAST analysis revealed the presence of *R. monacensis* in 14 *Ix. ricinus* and 35 *Ix. inopinatus* specimens, and *R. helvetica* in 6 *Ix. ricinus* and 6 *Ix. inopinatus* specimens (Tables 5, 7). In addition, all *Rh. sanguineus* s.l. ticks were positive for *R. massiliae* (Table 7).

**Figure 4:**
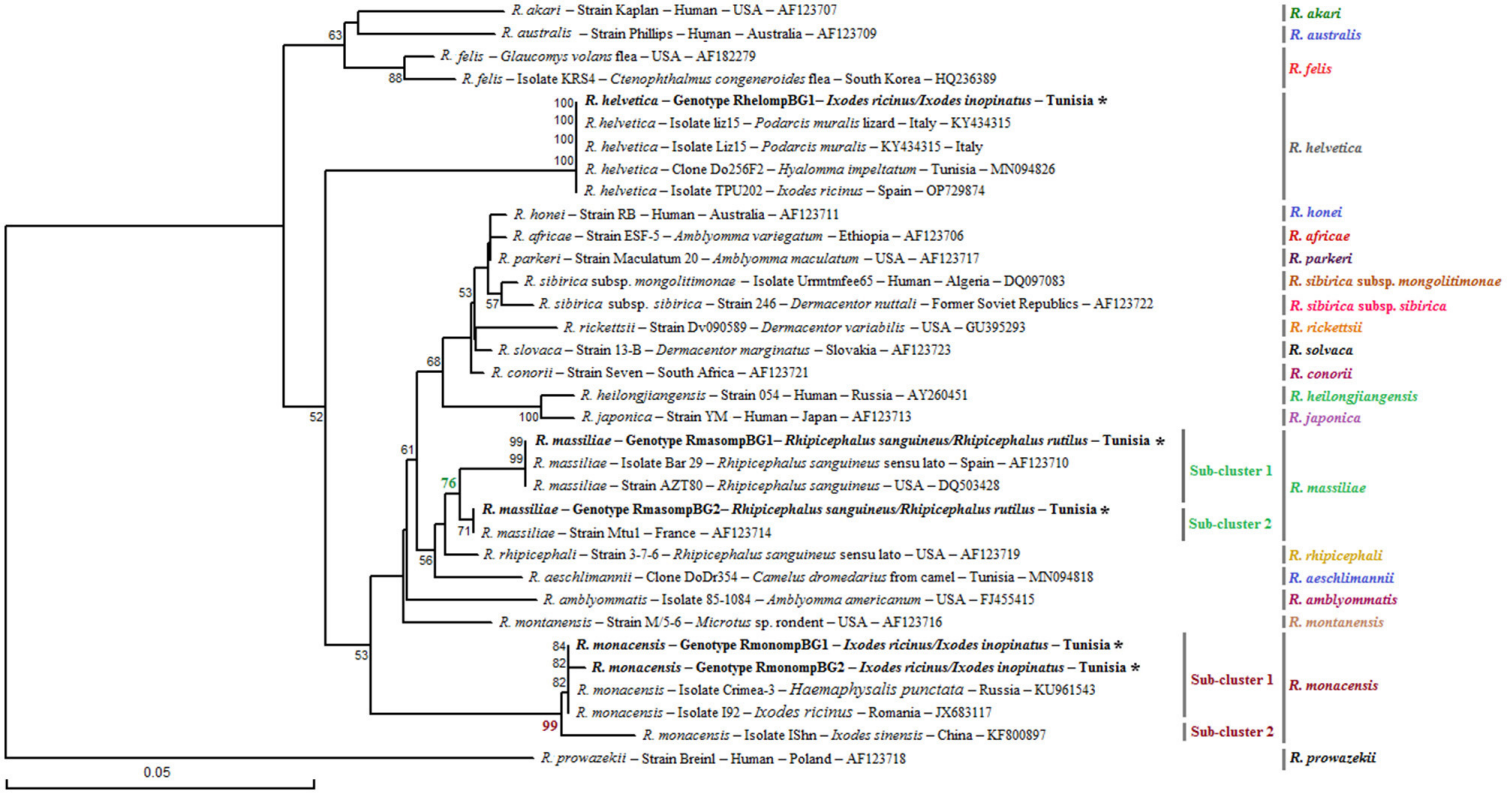
Phylogenetic tree of *Rickettsia* species based on partial *ompB* sequences (382 bp) of *Rickettsia* spp. obtained in this study, along with selected representative sequences of the genus *Rickettsia*. Bootstrap values from 1,000 replicates are shown above the branches (only values above 50% are displayed). Partial *ompB* sequences representing the different *Rickettsia* genotypes identified in this study are shown in bold and marked with an asterisk. Information on the host or vector, genotype, strain or isolate name, country of origin, and GenBank accession number is provided. A partial *ompB* sequence from *Rickettsia prowazekii* was included as an outgroup.

The genetic diversity analysis carried out using DnaSP version 5.10.01 software on a 382 bp of the *ompB* gene made it possible to identify two genotypes for *R. monacensis*, named RmonompBG1 and RmonompBG2, both found in *Ix. ricinus* (14 specimens) and *Ix. inopinatus* (35 specimens), with genetic diversity equal to 0.080. The GC rate was 51.6%. The nucleotide diversity (Pi) and average number of nucleotide differences (k) were estimated, respectively, at 0.00021 and 0.080 by noting the presence of two mutational positions between the two different revealed genotypes, sharing 99.74% nucleotide similarity (Table 4). RmonompBG1 and RmonompBG2 genotypes clustered with an isolate from *Ix. ricinus* in Romania (GenBank accession number: JX631117) and *Haemaphysalis punctata* in Russia (GenBank accession number: KU961543) (Figure 4).

The sequence alignment of *R. helvetica* revealed a single genotype named RhelompBG1 isolated from specimens belonging to *Ix. ricinus* and *Ix. inopinatus* (Tables 5, 6). This genotype was found identical to three previously reported *ompB* isolates: clone Do256F2 from a *Hy. impeltatum* tick in Tunisia (GenBank accession number: MN094826), isolate TPU202 from an *Ix. ricinus* tick in Spain (GenBank accession number: OP729874), and isolate liz15 detected in *Podarcis muralis* from Italy (GenBank accession number: KY434315) (Figure 4).

Additionally, the alignment of partial sequences belonging to *R. massiliae* made it possible to select two genotypes named RmasompBG1 and RmasompBG2 infecting six *Rh. sanguineus* s.l. specimens, with genetic diversity equal to 0.429. The GC rate was 50.6%. The nucleotide diversity (Pi) and average number of nucleotide differences (k) were estimated, respectively, at 0.00561 and 2.143 by noting the presence of two mutational positions between the two different revealed genotypes, sharing 98.69% nucleotide similarity (Tables 4, 7). The genotype RmasompBG1, identified in six *Rh. sanguineus* s.l. specimens was found identical to isolates from *Rh. sanguineus* s.l. in Spain (GenBank accession number: AF123710) and the USA (GenBank accession number: DQ503428). However, the second genotype RmasompBG2, detected in a single *Rh. sanguineus* specimen, was found identical to a pathogenic *R. massiliae* isolate previously reported in France (GenBank accession number: AF123714) (Figure 4).

Phylogenetic analysis, based on the alignment of *ompB* genotypes with reference sequences from GenBank, revealed distinct clusters corresponding to the three identified *Rickettsia* species (Figure 4). The *R. monacensis* cluster comprised two subclusters with a node robustness equal to 99%. The first subcluster included genotypes RmonompBG1 and RmonompBG2, which grouped with isolates from *Ix. ricinus* in Romania (GenBank accession number: JX631117) and *Haemaphysalis punctata* in Russia (GenBank accession number: KU961543). The second subcluster consisted solely of isolate IShn from *Ix. sinensis* in China (GenBank accession number: KF800897) (Figure 4). *R. helvetica* genotype RhelompBG1 formed a single cluster with previously reported isolates from *Hy. impeltatum* in Tunisia (GenBank accession number: MN094826), *Ix. ricinus* in Spain (GenBank accession number: OP729874), and *Podarcis muralis* in Italy (GenBank accession number: KY434315) (Figure 4). Besides, the *R. massiliae* cluster was divided into two closely related sub-clusters with a node robustness equal to 76%. Genotype RmasompBG1 grouped with isolates from *Rh. sanguineus* s.l. in Spain (GenBank accession number: AF123710) and the USA (GenBank accession number: DQ503428), while genotype RmasompBG2 clustered with the pathogenic strain Mtu1 from France (GenBank accession number: AF123714) (Figure 4).

#### 3.4.2 *Rickettsia* spp. *ompA* partial sequences

The analysis of *ompA* partial sequences confirmed the presence of *R. monacensis* and *R. Massiliae* (Tables 5, 6). The results showed that 9 *Ix. ricinus* and 16 *Ix. inopinatus* ticks tested positive for *R. monacensis*. However, 8 specimens of *Rh. sanguineus* were found positive for *R. massiliae* (Tables 5–7).

**Figure 5:**
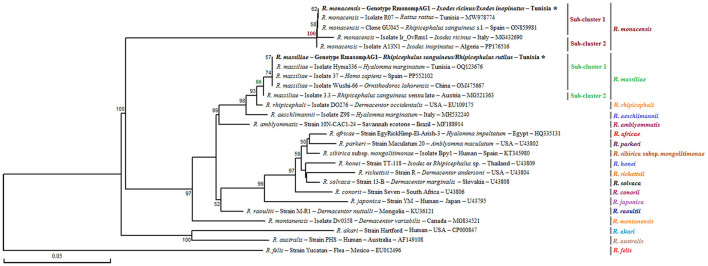
Neighbor-Joining tree based on the alignment of partial *ompA* sequences (490 bp), showing newly obtained sequences from ticks collected in vegetation. Bootstrap values from 1,000 replicates are shown at each node (only values above 50% are displayed). *Rickettsia* spp. genotypes identified in this study are shown in bold and marked with an asterisk. Information on the host or vector, genotype, strain or isolate name, country of origin, and GenBank accession number is provided. A partial *ompA* sequence from *Rickettsia felis* was included as an outgroup.

The alignment of sequences isolated for *R. monacensis* allowed us to select a single genotype named RmonompAG1 infecting 9 *Ix. ricinus* and 16 *Ix. inopinatus*. This genotype was found to be identical to isolates from *Rattus rattus* in Tunisia (GenBank accession number: MW978774) and *Rh. sanguineus* s.l in Spain (GenBank accession number: ON859981) (Figure 5). Furthermore, the sequences belonging to *R. massiliae* infecting eight *Rh. sanguineus* s.l. tick specimens allowed us to select a single genotype named RmasompAG1. The BLAST analysis showed that this genotype was identical to three previously reported isolates from *Hy. marginatum* in Tunisia (GenBank accession number: OQ123676), *Homo sapiens* in Spain (GenBank accession number: PP552102) and *Ornithodoros lahorensis* in China (GenBank accession number: OM475667) (Figure 5).

Phylogenetic analysis of the *ompA* gene, based on the alignment of our Tunisian genotypes with reference sequences from GenBank, revealed several distinct clusters (Figure 5). The *R. monacensis* cluster was divided into two sub-clusters with a node robustness equal to 100%. The genotype RmonompAG1 identified in this study grouped within the first sub-cluster, alongside isolates from *Rattus rattus* in Tunisia (isolate07; GenBank accession number: MW978774) and from *Rh. sanguineus* s.l. in Spain (clone GU045; GenBank accession number: ON859981) (Figure 5). The second sub-cluster comprised isolates Ir_OVrmx1 from *Ix. ricinus* in Italy (GenBank accession number: MG432690) and A13N1 from *Ix. inopinatus* in Algeria (GenBank accession number: PP176516) (Figure 5). On the other hand, the *R. massiliae* cluster also formed two subclusters with a node robustness equal to 86%. The genotype identified in this study (RmasompAG1) clustered within the first sub-cluster, together with isolates from *Hy. marginatum* in Tunisia (GenBank accession number: OQ123676), a human isolate from Spain (GenBank accession number: PP552102), and *Ornithodoros lahorensis* in China (GenBank accession number: OM475667) (Figure 5).

#### 3.4.3 *Rickettsia* spp. *gltA* partial sequences

**Figure 6:**
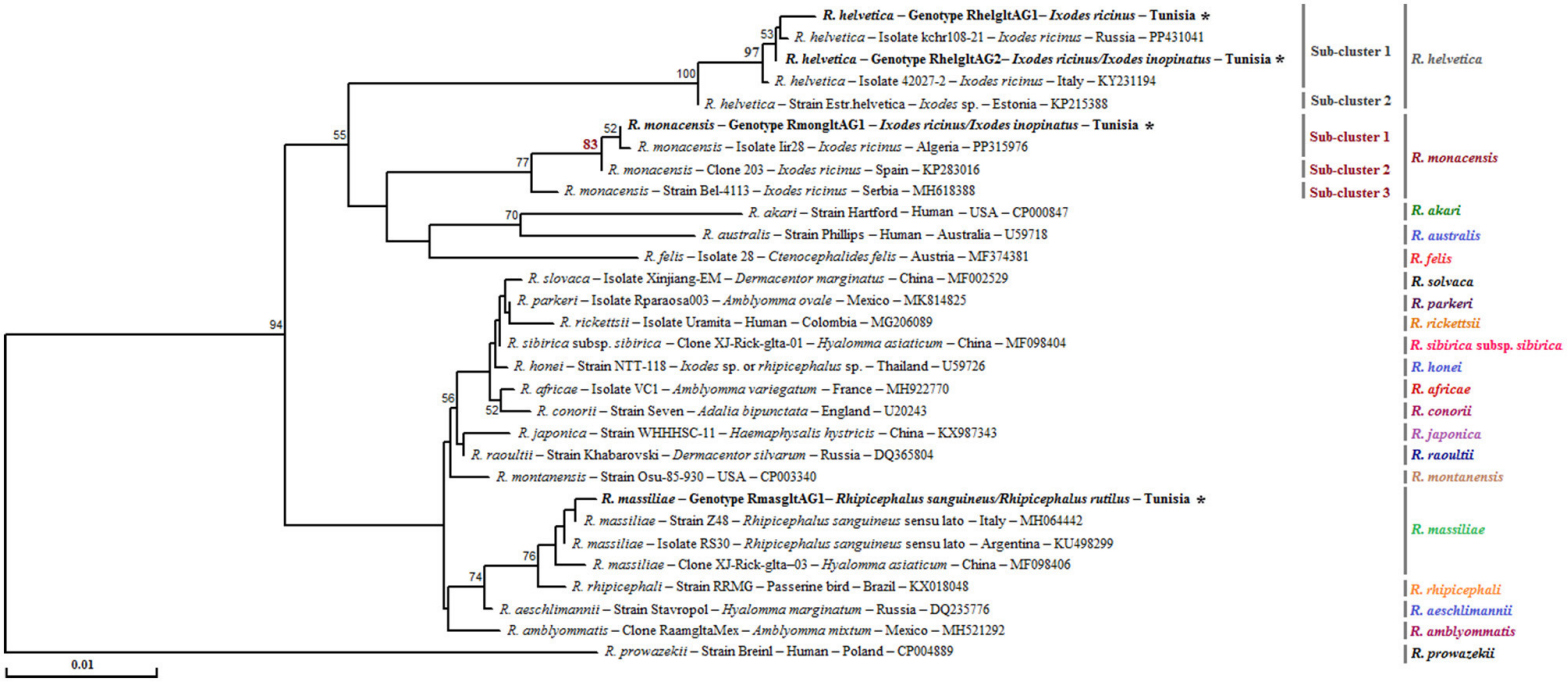
Neighbor-Joining tree based on the alignment of partial *gltA* sequences (341 bp), showing newly obtained sequences from ticks collected in vegetation. Bootstrap values from 1,000 replicates are shown at each node (only values above 50% are displayed). *Rickettsia* spp. genotypes identified in this study are shown in bold and marked with an asterisk. Information on the host or vector, genotype, strain or isolate name, country of origin, and GenBank accession number is provided. A partial *ompA* sequence from *Rickettsia prowazekii* was included as an outgroup.

Sequencing of a 341 bp fragment of the *gltA* gene, which corresponds to the 381 bp amplified sequence excluding the forward and reverse primer sequences, revealed infections with *R. monacensis, R. helvetica* and *R. massiliae* (Tables 1–7). BLAST analysis confirmed the infection of 8 *Ix. ricinus* and 10 *Ix. inopinatus* with *R. monacensis* (Tables 5, 6). In addition, 4 *Ix. ricinus* and 5 *Ix. inopinatus* ticks tested positive for *R. helvetica* (Tables 5, 6). However, two *Rh. rutilus* ticks were found to be infected with *R. massiliae* along with 5 *Rh. sanguineus* tick specimens (Table 7). Sequence alignment of *R. monacensis* revealed a single genotype named Rmon*gltA*G1 infecting 8 and 10 specimens belonging, respectively, to *Ix. ricinus* and *Ix. inopinatus* (Tables 5, 6). This genotype was found to be identical to the *R. monacensis* isolate IIR28 infecting one *Ix. ricinus* from Algeria (GenBank accession number: PP315976) (Figure 6). In addition, the sequence alignment of *R. helvetica* isolates identified two genotypes named Rhel*gltA*G1 and Rhel*gltA*G2, infecting 4 *Ix. ricinus* and 5 *Ix. inopinatus* specimens, with genetic diversity equal to 0.222 (Tables 4–6). The GC content of the *gltA* sequences was 48.4%. The nucleotide diversity (Pi) and average number of nucleotide differences (k) were estimated at 0.00065 and 0.222, respectively, by noting the presence of two mutational positions between the two different revealed genotypes, sharing 99.71% nucleotide similarity (Table 4). These genotypes shared 99.71% and 100% genetic similarity, respectively, with an isolate KChR108-21 of *R. helvetica* from *Ix. ricinus* in Russia (GenBank accession number: PP431041). Alignment of *R. massilae* sequences revealed a single genotype named Rmas*gltA*G1 identified in two *Rh. rutilus* and five *Rh. sanguineus*, respectively (Table 7). This genotype was found to be identical to the *R. monacensis* isolate IIR28 infecting one *Ix. ricinus* from Algeria (GenBank accession number: PP315976) (Figure 6).

Phylogenetic analysis of the *gltA* gene revealed that the *R. monacensis* cluster comprised three sub-clusters with a node robustness equal to 83% (Figure 6). The genotype RmongltAG1 identified in this study grouped within the first sub-cluster, along with isolate lir28 from *Ix. ricinus* in Algeria (GenBank accession number: PP315976) (Figure 6). For *R. helvetica*, both genotypes RhelgltAG1 and RmongltAG2 clustered within the first sub-cluster with an isolate from *Ix. ricinus* in Russia (GenBank accession number: PP431041) (Figure 6). Additionally, the *R. massiliae* cluster was homogeneous, with a single genotype (RmasgltAG1) detected in this study. This genotype clustered with several previously reported *R. massiliae* isolates from *Rh. sanguineus* s.l specimens collected from several countries, as well as with an isolate from *Hyalomma asiaticum* (GenBank accession numbers: MH064442, KU498299, and MF098406) (Figure 6).

## 4 Discussion

Hard ticks (Acari: Ixodidae) are among the most significant arthropod vectors of *Rickettsia* spp. (Estrada-Peña et al., 2021). Several species within the genera *Hyalomma, Amblyomma, Rhipicephalus, Ixodes, Haemaphysalis*, and *Dermacentor* have been confirmed as competent vectors of these bacteria (Ammerman et al., 2004; Parola et al., 2005; Fournier and Raoult, 2019). Yet, in Tunisia, and more broadly across North Africa, data on the circulation of *Rickettsia* remain surprisingly limited. The present study contributes to filling this gap by conducting a descriptive investigation at a single location in Jouza district, Beja governorate, northwestern Tunisia. Ticks were collected from both vegetation and a red fox (*V. vulpes*) and their associated *Rickettsia* species were molecularly characterized.

Tick identification based on both morphological and molecular criteria confirmed the presence of five species: *Ix. inopinatus, Ix. ricinus, Ix. hexagonus, Rh. sanguineus* (temperate lineage), and *Rh. rutilus*. The detection of *Ix. ricinus* and *Ix. inopinatus* supports previous studies reporting their sympatric distribution in humid, forested regions of northern Tunisia (Younsi et al., 2019; Elati et al., 2022). The >99% identity of our sequences with European references confirms strong genetic relatedness between North African and European populations, likely reflecting shared Palearctic biogeographic patterns (Noureddine et al., 2011). Interestingly, *Ix. inopinatus*, initially described in southwestern Europe, has been increasingly detected across North Africa. In Algeria, (Mechouk et al. 2022) documented its wide habitat range, and (Daněk et al. 2024) identified all 149 *Ixodes* specimens in northeastern Algeria as *Ix. inopinatus*, suggesting ecological dominance or superior adaptation relative to *Ix. ricinus*. These findings raise important, yet unaddressed, ecological questions in the Tunisian context, particularly regarding niche partitioning, host preference, and how climate or land-use changes might be reshaping local species dynamics (Rosà et al., 2018; Horak et al., 2022; Hoch et al., 2024). Adding to this complexity, our phylogenetic analyses revealed substantial intra-specific variability in *Ixodes* spp., with 12 genotypes in *Ix. ricinus* and 10 in *Ix. inopinatus*. The presence of such high diversity in a single locality suggests multiple coexisting lineages shaped by host availability, microhabitat variation, or historical dispersal (Noureddine et al., 2011; Remesar et al., 2019). This is consistent with studies across the Mediterranean and Europe where similar patterns were interpreted as evidence of local structuring and possible cryptic speciation (Elati et al., 2022). In North Africa, the sympatric occurrence of genetically divergent *Ixodes* haplotypes may also signal introgression events or emerging species complexes, topics that call for deeper population-level resolution using high-throughput genomic tools.

Our study also marks the first molecular confirmation of *Ix. hexagonus* in Tunisia. Known for its nidicolous behavior and preference for hedgehogs, it also parasitizes a broad range of mammals, including foxes, mustelids, and domestic pets (Balti et al., 2021; Valentyna and Beata, 2024). Its detection on a red fox in Beja may reflect either an overlooked endemic presence or a subtle range expansion, masked by its endophilic lifestyle and under-sampling of wild hosts. In Algeria, this species was previously found on hedgehogs and dogs (Mechouk et al., 2022), and its widespread European distribution from the UK to Germany attests to its ecological plasticity (Abdullah et al., 2016; Cull et al., 2018; Kahl et al., 2022). In addition, the low mitochondrial 16S rRNA diversity observed in our specimens, all nymphs, may be linked to collection bias (single host), developmental constraints, or restricted dispersal associated with its lifestyle (Hornok et al., 2017). Furthermore, the high GC content and slow evolutionary rate of the 16S marker, especially in immature stages, could mask finer-scale variation (Boore, 1999; Estrada-Peña et al., 2017). Regarding the *Rh. sanguineus* s.l. complex, we confirmed the presence of both *Rh. sanguineus* (temperate lineage) and *Rh. rutilus*, consistent with recent findings in Tunisia and surrounding regions (Elati et al., 2018; Jomli et al., 2025; Senbill et al., 2024). Our phylogenetic analysis revealed that *Rh. sanguineus* clustered within sub-cluster 1 of *Rh. sanguineus* sensu stricto, while *Rh. rutilus* formed a distinct clade comprising three sub-clusters. Moreover, our genotype grouped with a Greek isolate (Ligda et al., 2025), reinforcing the unresolved taxonomy and complex genetic structure within the *Rhipicephalus* genus (Dantas-Torres et al., 2017; Estrada-Peña et al., 2017). This presence of host-specific or ecologically distinct genotypes suggests adaptive divergence possibly driven by geographic isolation or selective pressures which are mechanisms well recognized in tick evolution, but understudied in the Maghreb.

Infection prevalence by *Rickettsia* spp. reached 52.1% across all collected ticks. Among them, *Ix. ricinus* showed the highest infection rate (75.6%), comparable to levels reported in Germany (52.5%) and Poland (42.3%) (May and Strube, 2014; Dyczko et al., 2024). *Ix. inopinatus* also showed a high prevalence (67.4%), in agreement with findings from Algeria (81.9%) and Italy (31.2%) (Daněk et al., 2024). These results confirm the important role of *Ixodes* species in pathogen transmission in Tunisia and suggest that *Ix. inopinatus*, though less studied, might be as epidemiologically relevant as *Ix. ricinus*. On the other hand, *Ix. hexagonus* tested negative, possibly due to the nymphal stage of the specimens, which may limit pathogen acquisition and maintenance. This aligns with prior studies showing lower infection rates in immature ticks (Hartelt et al., 2004; Parola et al., 2005; Socolovschi et al., 2009). Additional sampling of adults is thus essential to clarify this species' potential role. Moreover, ticks of the *Rh. sanguineus* s.l. complex exhibited an overall infection rate of 37.2%, comparable to rates previously reported in *Rhipicephalus* ticks infesting small ruminants and cattle in Tunisia reported, respectively, by (Belkahia et al. 2021) and (Kratou et al. 2023). The detection of multiple genotypes in different host and environmental contexts suggests microevolutionary differentiation and possibly varying vectorial capacities (Gómez-Díaz et al., 2010; Jia et al., 2020; Ring et al., 2022).

Through sequencing of *ompB, ompA*, and *gltA* gene fragments, we identified *R. monacensis, R. helvetica*, and *R. massiliae*. *Rickettsia monacensis* was the most frequently detected species in *Ixodes* ticks from both vegetation and the red fox. This observation reinforces the role of *Ix. ricinus* and *Ix. inopinatus* as key vectors of these zoonotic agents, in line with previous reports from Europe and North Africa (Daněk et al., 2024). These results are consistent with European data, where *R. monacensis* predominates in southern regions such Bavaria (Germany) and Luxembourg, where *R. helvetica* predominated in *Ix. ricinus* populations (Reye et al., 2010; Schorn et al., 2011). In contrast, in southern and eastern Europe, *R. monacensis* was more commonly detected. For instance, studies from Spain have shown that the majority of *Ix. ricinus* specimens collected from vegetation or mammals were infected with *R. monacensis*, while *R. helvetica* was rarely identified (Márquez, 2008). A similar pattern was reported in Turkey, where *R. monacensis* showed significantly higher infection rates than *R. helvetica* (Gargili et al., 2012). These geographic differences in *Rickettsia* prevalence underscore the influence of environmental, ecological, and host-related factors on pathogen circulation within *Ixodes* populations. The high sequence similarity of our *R. monacensis* isolates with strains from Algeria, Spain, and even *Rattus rattus* in Tunisia suggests possible trans-Mediterranean circulation, perhaps facilitated by migratory birds or anthropogenic movements (Wasfi et al., 2021). Moreover, the detection of *R. helvetica*, especially in *Ix. ricinus*, and its close similarity to strains from the south of Tunisia, Spain, and Russia (Tomassone et al., 2017; Selmi et al., 2019; Rakov et al., 2024), reinforces its status as a widespread zoonotic agent in temperate zones. Several small mammal species and deer have been suggested as potential reservoirs (Špitalská et al., 2014), and the identification of *R. helvetica* in Tunisia raises questions about the local vertebrate hosts involved in its maintenance. On the other hand, *R. massiliae* was detected in *Rh. sanguineus* s.l. and *Rh. rutilus*, and sequences showed high similarity to isolates from France, Spain, and the United States (Roux and Raoult, 2000; Eremeeva et al., 2006), including strains previously reported from cattle, dogs, humans, and even *Ornithodoros lahorensis* in China (Vieira Lista et al., 2024). This confirms its broad host range and cosmopolitan distribution (Khrouf et al., 2013; Selmi et al., 2019; Belkahia et al., 2021; Kratou et al., 2023). Given the presence of pathogenic strains and the close proximity of these ticks to human and domestic animal populations, the potential public health risk cannot be overlooked. Given the detection of zoonotic *Rickettsia* species, including *R. monacensis, R. helvetica*, and *R. massiliae*, this study also highlights a tangible risk of pathogen spillover into human populations. These bacteria have been implicated in human febrile illnesses across Europe and the Mediterranean basin, with documented clinical cases associated with *R. massiliae* in France and the USA (Beati and Raoult, 1993; Eremeeva et al., 2006), *R. monacensis* in Spain and Germany (Márquez, 2008; Blanton, 2019), and *R. helvetica* in Switzerland and Sweden (Nilsson et al., 1999; Fournier, 2000). The close phylogenetic proximity of our sequences to those linked to human infections underscores the need to consider these pathogens in the differential diagnosis of febrile syndromes and to enhance integrated tick-borne disease surveillance in Tunisia under a *One Health* framework (Parola et al., 2013; Onyiche et al., 2022).

This study provides valuable baseline data on the diversity of *Ixodes* and *Rhipicephalus* ticks and their associated *Rickettsia* (spotted fever group) at an ecologically relevant site in Jouza, in the Beja Governorate, northwestern Tunisia, a region belonging to the subhumid bioclimatic zone favorable to these tick genera. However, several limitations should be noted. First, the focus on a single site limits the generalizability of the findings across Tunisia. This targeted descriptive approach was adopted as a pioneering study aimed at precisely characterizing local tick species and their *Rickettsia* diversity using molecular methods, including 16S rRNA sequencing of all tick samples and most amplicons of the three markers used to identify and characterize the revealed *Rickettsia* species. Future studies extending to multiple sites and diverse bioclimatic zones will be essential for providing broader epidemiological insights. Second, the host range sampled was primarily limited to ticks collected from vegetation, with the opportunistic inclusion of a single heavily infested red fox (*V. vulpes*). While this unexpected finding adds originality and highlights the potential role of wildlife in tick ecology, a more systematic inclusion of diverse wild and domestic hosts is needed to better understand host-pathogen interactions and reservoir dynamics. Third, some tick species, such as *Rh. rutilus*, were represented by relatively few specimens, which limits statistical power and phylogenetic resolution for these taxa. Increasing sample sizes for less common species in future research will strengthen these analyses. Fourth, molecular characterization relied on partial sequences of key genes (*ompA, ompB*, and *gltA*), providing robust initial insights into species and strain diversity. Nonetheless, whole-gene or whole-genome sequencing approaches would yield higher resolution of genetic variation and evolutionary relationships. Finally, this study focused exclusively on *Rickettsia* species, limiting the understanding of the full spectrum of tick-borne pathogens in the region. Incorporating screening for other important pathogens such as *Borrelia* and *Anaplasma* in future studies would provide a more comprehensive view of tick-borne disease ecology and co-infection patterns. Despite these limitations, this pioneering study offers critical baseline information and establishes a framework for more expansive epidemiological research across northern Tunisia.

## 5 Conclusion

This study confirms the presence of ticks belonging to the *Ix. ricinus* complex (*Ix. ricinus* and *Ix. inopinatus*), *Ix. hexagonus*, and the *Rh. sanguineus* s.l. complex (*Rh. sanguineus* (temperate lineage) and *Rh. rutilus*) in northwestern Tunisia. It highlights the presence of *R. monacensis, R. helvetica*, and *R. massiliae* in these ticks, emphasizing their role as vectors for spotted fever group rickettsiae. These findings enhance our understanding of the regional circulation of *Rickettsia* spp. in North Africa and underscore the need for ongoing epidemiological and molecular surveillance to evaluate their zoonotic potential. Such efforts are crucial for informing public health strategies and reducing the risk of tick-borne rickettsioses in the Mediterranean region.

## Data Availability

The datasets presented in this study can be found in online repositories. The names of the repository/repositories and accession number(s) can be found in the article/Supplementary material.
