# Supplementary material for: Molecular identification, genotyping and phylogenetic analysis of Ixodes and Rhipicephalus ticks and their associated spotted fever group Rickettsia species from a single location in northern Tunisia
Source: Front Microbiol. 2025 Aug 14;16:1644524. doi: 10.3389/fmicb.2025.1644524 (PMC12391194; doi:10.3389/fmicb.2025.1644524)
Supplement: Supplementary file 1 [file Table_1.docx]

| Sample | Morp. Id. | Host or environment | *Rickettsia* (+/-) | BLAST^1^ (GenBank^2^, Genotype) |
| --- | --- | --- | --- | --- |
| Ixric68 | *Ix. ricinus* complex | Vegetation | *Rickettsia +* | 100% *Ixodes ricinus* (PV018138, Ixric16SG6) |
| Ixric88 | *Ix. ricinus* complex | Vegetation | *Rickettsia +* | 100% *Ixodes ricinus* (PV018139, Ixric16SG6) |
| Ixric100 | *Ix. ricinus* complex | Vegetation | *Rickettsia +* | 100% *Ixodes ricinus* (PV018140, Ixric16SG6) |
| Ixric157 | *Ix. ricinus* complex | *Vulpes vulpes* | *Rickettsia +* | 100% *Ixodes ricinus* (PV018141, Ixric16SG6) |
| Ixric16 | *Ix. ricinus* complex | Vegetation | *Rickettsia -* | 100% *Ixodes ricinus* (PV018142, Ixric16SG2) |
| Ixric17 | *Ix. ricinus* complex | Vegetation | *Rickettsia -* | 100% *Ixodes ricinus* (PV018143, Ixric16SG6) |
| Ixric32 | *Ix. ricinus* complex | Vegetation | *Rickettsia -* | 100% *Ixodes ricinus* (PV018144, Ixric16SG2) |
| Ixric81 | *Ix. ricinus* complex | Vegetation | *Rickettsia -* | 100% *Ixodes ricinus* (PV018145, Ixric16SG6) |
| Ixric113 | *Ix. ricinus* complex | *Vulpes vulpes* | *Rickettsia -* | 100% *Ixodes ricinus* (PV018146, Ixric16SG6) |
| Ixric115 | *Ix. ricinus* complex | *Vulpes vulpes* | *Rickettsia -* | 100% *Ixodes ricinus* (PV018147, Ixric16SG6) |
| Ixric119 | *Ix. ricinus* complex | *Vulpes vulpes* | *Rickettsia -* | 100% *Ixodes ricinus* (PV018148, Ixric16SG6) |
| Ixric128 | *Ix. ricinus* complex | *Vulpes vulpes* | *Rickettsia -* | 100% *Ixodes ricinus* (PV018149, Ixric16SG6) |
| Ixric139 | *Ix. ricinus* complex | *Vulpes vulpes* | *Rickettsia -* | 100% *Ixodes ricinus* (PV018150, Ixric16SG12) |
| Ixric152 | *Ix. ricinus* complex | *Vulpes vulpes* | *Rickettsia -* | 100% *Ixodes ricinus* (PV018151, Ixric16SG6) |
| Ixric163 | *Ix. ricinus* complex | *Vulpes vulpes* | *Rickettsia -* | 100% *Ixodes ricinus* (PV018152, Ixric16SG2) |

**Supplementary file 1**: Designation, information on the origins, infection status by *Rickettsia* spp., and mitochondrial 16S rRNA genotypes of remaining Tunisian isolates of *Ixodes ricinus* ticks

Abbreviations: *Rickettsia* (+/-): Positive or negative to *Rickettsia* spp. *ompB* PCR, ^1^ BLAST analysis for mitochondrial 16S rRNA partial sequence of ticks; ^2^ GenBank accession number.
