# Supplementary material for: Molecular identification, genotyping and phylogenetic analysis of Ixodes and Rhipicephalus ticks and their associated spotted fever group Rickettsia species from a single location in northern Tunisia
Source: Front Microbiol. 2025 Aug 14;16:1644524. doi: 10.3389/fmicb.2025.1644524 (PMC12391194; doi:10.3389/fmicb.2025.1644524)
Supplement: Supplementary file 2 [file Table_2.docx]

| Sample | Morp. Id. | Host or environment | *Rickettsia* (+/-) | BLAST^1^ (GenBank^2^, Genotype) |
| --- | --- | --- | --- | --- |
| Ixinp44 | *Ix. ricinus* complex | Vegetation | *Rickettsia +* | 99.6% *Ixodes inopinatus* (PV018208, Ixino16SG1) |
| Ixinp47 | *Ix. ricinus* complex | Vegetation | *Rickettsia +* | 99.6% *Ixodes inopinatus* (PV018209, Ixino16SG1) |
| Ixinop73 | *Ix. ricinus* complex | Vegetation | *Rickettsia +* | 99.6% *Ixodes inopinatus* (PV018210, Ixino16SG1) |
| Ixinop105 | *Ix. ricinus* complex | Vegetation | *Rickettsia +* | 99.6% *Ixodes inopinatus* (PV018211, Ixino16SG7) |
| Ixinop26 | *Ix. ricinus* complex | Vegetation | *Rickettsia +* | 99.6% *Ixodes inopinatus* (PV018212, Ixino16SG1) |
| Ixinp1 | *Ix. ricinus* complex | Vegetation | *Rickettsia -* | 99.6% *Ixodes inopinatus* (PV018213, Ixino16SG1) |
| Ixinp2 | *Ix. ricinus* complex | Vegetation | *Rickettsia -* | 99.6% *Ixodes inopinatus* (PV018214, Ixino16SG1) |
| Ixinp9 | *Ix. ricinus* complex | Vegetation | *Rickettsia -* | 99.6% *Ixodes inopinatus* (PV018215, Ixino16SG1) |
| Ixinp14 | *Ix. ricinus* complex | Vegetation | *Rickettsia -* | 99.6% *Ixodes inopinatus* (PV018216, Ixino16SG1) |
| Ixinp19 | *Ix. ricinus* complex | Vegetation | *Rickettsia -* | 99.6% *Ixodes inopinatus* (PV018217, Ixino16SG8) |
| Ixinp28 | *Ix. ricinus* complex | Vegetation | *Rickettsia -* | 99.6% *Ixodes inopinatus* (PV018218, Ixino16SG1) |
| Ixinp40 | *Ix. ricinus* complex | Vegetation | *Rickettsia -* | 99.6% *Ixodes inopinatus* (PV018219, Ixino16SG1) |
| Ixinp41 | *Ix. ricinus* complex | Vegetation | *Rickettsia -* | 99.6% *Ixodes inopinatus* (PV018220, Ixino16SG1) |
| Ixinp53 | *Ix. ricinus* complex | Vegetation | *Rickettsia -* | 99.6% *Ixodes inopinatus* (PV018221, Ixino16SG1) |
| Ixinp69 | *Ix. ricinus* complex | Vegetation | *Rickettsia -* | 99.6% *Ixodes inopinatus* (PV018222, Ixino16SG1) |
| Ixinp80 | *Ix. ricinus* complex | Vegetation | *Rickettsia -* | 99.6% *Ixodes inopinatus* (PV018223, Ixino16SG1) |
| Ixinp97 | *Ix. ricinus* complex | Vegetation | *Rickettsia -* | 99.6% *Ixodes inopinatus* (PV018224, Ixino16SG1) |
| Ixinp103 | *Ix. ricinus* complex | Vegetation | *Rickettsia -* | 99.6% *Ixodes inopinatus* (PV018225, Ixino16SG1) |
| Ixinp114 | *Ix. ricinus* complex | *Vulpes vulpes* | *Rickettsia -* | 99.6% *Ixodes inopinatus* (PV018226, Ixino16SG1) |
| Ixinp120 | *Ix. ricinus* complex | *Vulpes vulpes* | *Rickettsia -* | 99.6% *Ixodes inopinatus* (PV018227, Ixino16SG1) |
| Ixinp122 | *Ix. ricinus* complex | *Vulpes vulpes* | *Rickettsia -* | 99.6% *Ixodes inopinatus* (PV018228, Ixino16SG1) |
| Ixinp126 | *Ix. ricinus* complex | *Vulpes vulpes* | *Rickettsia -* | 99.6% *Ixodes inopinatus* (PV018229, Ixino16SG1) |
| Ixinp127 | *Ix. ricinus* complex | *Vulpes vulpes* | *Rickettsia -* | 99.6% *Ixodes inopinatus* (PV018230, Ixino16SG9) |
| Ixinp132 | *Ix. ricinus* complex | *Vulpes vulpes* | *Rickettsia -* | 99.6% *Ixodes inopinatus* (PV018231, Ixino16SG1) |
| Ixinp134 | *Ix. ricinus* complex | *Vulpes vulpes* | *Rickettsia -* | 99.6% *Ixodes inopinatus* (PV018232, Ixino16SG1) |
| Ixinp154 | *Ix. ricinus* complex | *Vulpes vulpes* | *Rickettsia -* | 99.6% *Ixodes inopinatus* (PV018233, Ixino16SG1) |
| Ixinp156 | *Ix. ricinus* complex | *Vulpes vulpes* | *Rickettsia -* | 99.6% *Ixodes inopinatus* (PV018234, Ixino16SG1) |
| Ixinp160 | *Ix. ricinus* complex | *Vulpes vulpes* | *Rickettsia -* | 99.6% *Ixodes inopinatus* (PV018235, Ixino16SG1) |
| Ixinp164 | *Ix. ricinus* complex | *Vulpes vulpes* | *Rickettsia -* | 99.6% *Ixodes inopinatus* (PV018236, Ixino16SG1) |
| Ixinp173 | *Ix. ricinus* complex | *Vulpes vulpes* | *Rickettsia -* | 99.6% *Ixodes inopinatus* (PV018237, Ixino16SG1) |
| Ixinp176 | *Ix. ricinus* complex | *Vulpes vulpes* | *Rickettsia -* | 99.6% *Ixodes inopinatus* (PV018238, Ixino16SG1) |
| Ixinp177 | *Ix. ricinus* complex | *Vulpes vulpes* | *Rickettsia -* | 99.6% *Ixodes inopinatus* (PV018239, Ixino16SG1) |
| Ixinp168 | *Ix. ricinus* complex | *Vulpes vulpes* | *Rickettsia -* | 100% *Ixodes inopinatus* (PV018240, Ixino16SG10) |

**Supplementary file 2**: Designation, information on the origins, infection status by *Rickettsia* spp., and mitochondrial 16S rRNA genotypes of remaining Tunisian isolates of *Ixodes* *inopinatus* ticks

Abbreviations: *Rickettsia* (+/-): Positive or negative to *Rickettsia* spp. *ompB* PCR, ^1^ BLAST analysis for mitochondrial 16S rRNA partial sequence of ticks; ^2^ GenBank accession number.
