# Supplementary material for: Molecular identification, genotyping and phylogenetic analysis of Ixodes and Rhipicephalus ticks and their associated spotted fever group Rickettsia species from a single location in northern Tunisia
Source: Front Microbiol. 2025 Aug 14;16:1644524. doi: 10.3389/fmicb.2025.1644524 (PMC12391194; doi:10.3389/fmicb.2025.1644524)
Supplement: Supplementary file 3 [file Table_3.docx]

**Supplementary file 3:** Designation, information on the origins, infection status by *Rickettsia* spp., and mitochondrial 16S rRNA genotypes of Tunisian isolates of of *Ixodes hexagonus* ticks

| Sample | Morp. Id. | Host | *Rickettsia* (+/-) | BLAST^1^ (GenBank^2^, Genotype) |
| --- | --- | --- | --- | --- |
| Ixhex108 | *Ixodes* sp. | *Vulpes vulpes* | *Rickettsia -* | 98.9% *Ixodes hexagonus* (PV018241, Ixhex16SG1) |
| Ixhex109 | *Ixodes* sp. | *Vulpes vulpes* | *Rickettsia -* | 98.9% *Ixodes hexagonus* (PV018242, Ixhex16SG1) |
| Ixhex111 | *Ixodes* sp. | *Vulpes vulpes* | *Rickettsia -* | 98.9% *Ixodes hexagonus* (PV018243, Ixhex16SG1) |
| Ixhex112 | *Ixodes* sp. | *Vulpes vulpes* | *Rickettsia -* | 98.9% *Ixodes hexagonus* (PV018244, Ixhex16SG1) |
| Ixhex116 | *Ixodes* sp. | *Vulpes vulpes* | *Rickettsia -* | 98.9% *Ixodes hexagonus* (PV018245, Ixhex16SG1) |
| Ixhex117 | *Ixodes* sp. | *Vulpes vulpes* | *Rickettsia -* | 98.9% *Ixodes hexagonus* (PV018246, Ixhex16SG1) |
| Ixhex118 | *Ixodes* sp. | *Vulpes vulpes* | *Rickettsia -* | 98.9% *Ixodes hexagonus* (PV018247, Ixhex16SG1) |
| Ixhex135 | *Ixodes* sp. | *Vulpes vulpes* | *Rickettsia -* | 98.9% *Ixodes hexagonus* (PV018248, Ixhex16SG1) |
| Ixhex136 | *Ixodes* sp. | *Vulpes vulpes* | *Rickettsia -* | 98.9% *Ixodes hexagonus* (PV018249, Ixhex16SG1) |
| Ixhex137 | *Ixodes* sp. | *Vulpes vulpes* | *Rickettsia -* | 98.9% *Ixodes hexagonus* (PV018250, Ixhex16SG1) |
| Ixhex138 | *Ixodes* sp. | *Vulpes vulpes* | *Rickettsia -* | 98.9% *Ixodes hexagonus* (PV018251, Ixhex16SG1) |
| Ixhex140 | *Ixodes* sp. | *Vulpes vulpes* | *Rickettsia -* | 98.9% *Ixodes hexagonus* (PV018252, Ixhex16SG1) |
| Ixhex142 | *Ixodes* sp. | *Vulpes vulpes* | *Rickettsia -* | 98.9% *Ixodes hexagonus* (PV018253, Ixhex16SG1) |
| Ixhex143 | *Ixodes* sp. | *Vulpes vulpes* | *Rickettsia -* | 98.9% *Ixodes hexagonus* (PV018254, Ixhex16SG1) |
| Ixhex144 | *Ixodes* sp. | *Vulpes vulpes* | *Rickettsia -* | 98.9% *Ixodes hexagonus* (PV018255, Ixhex16SG1) |
| Ixhex145 | *Ixodes* sp. | *Vulpes vulpes* | *Rickettsia -* | 98.9% *Ixodes hexagonus* (PV018256, Ixhex16SG1) |
| Ixhex146 | *Ixodes* sp. | *Vulpes vulpes* | *Rickettsia -* | 98.9% *Ixodes hexagonus* (PV018257, Ixhex16SG1) |
| Ixhex147 | *Ixodes* sp. | *Vulpes vulpes* | *Rickettsia -* | 98.9% *Ixodes hexagonus* (PV018258, Ixhex16SG1) |
| Ixhex148 | *Ixodes* sp. | *Vulpes vulpes* | *Rickettsia -* | 98.9% *Ixodes hexagonus* (PV018259, Ixhex16SG1) |
| Ixhex149 | *Ixodes* sp. | *Vulpes vulpes* | *Rickettsia -* | 98.9% *Ixodes hexagonus* (PV018260, Ixhex16SG1) |
| Ixhex150 | *Ixodes* sp. | *Vulpes vulpes* | *Rickettsia -* | 98.9% *Ixodes hexagonus* (PV018261, Ixhex16SG1) |
| Ixhex151 | *Ixodes* sp. | *Vulpes vulpes* | *Rickettsia -* | 98.9% *Ixodes hexagonus* (PV018262, Ixhex16SG1) |
| Ixhex153 | *Ixodes* sp. | *Vulpes vulpes* | *Rickettsia -* | 98.9% *Ixodes hexagonus* (PV018263, Ixhex16SG1) |
| Ixhex185 | *Ixodes* sp. | *Vulpes vulpes* | *Rickettsia -* | 98.9% *Ixodes hexagonus* (PV018264, Ixhex16SG1) |

Abbreviations: *Rickettsia* (+/-): Positive or negative to *Rickettsia* spp. *ompB* PCR, ^1^ BLAST analysis for mitochondrial 16S rRNA partial sequence of ticks; ^2^ GenBank accession number.
