# Supplementary material for: Molecular identification, genotyping and phylogenetic analysis of Ixodes and Rhipicephalus ticks and their associated spotted fever group Rickettsia species from a single location in northern Tunisia
Source: Front Microbiol. 2025 Aug 14;16:1644524. doi: 10.3389/fmicb.2025.1644524 (PMC12391194; doi:10.3389/fmicb.2025.1644524)
Supplement: Supplementary file 4 [file Table_4.docx]

| Sample | Morp. Id. | Host | *Rickettsia* (+/-) | BLAST^1^ (GenBank^2^, Genotype) |
| --- | --- | --- | --- | --- |
| Rhrut75 | *Rh. sanguineus* s.l. | Vegetation | *Rickettsia -* | 100% *Rhipicephalus rutilus* (PV018269, Rhrut16SG1) |
| Rhsan5 | *Rh. sanguineus* s.l. | Vegetation | *Rickettsia +* | 100% *Rhipicephalus sanguineus* (PV018278, Rhsan16SG1) |
| Rhsan60 | *Rh. sanguineus* s.l. | Vegetation | *Rickettsia +* | 100% *Rhipicephalus sanguineus* (PV018279, Rhsan16SG1) |
| Rhsan61 | *Rh. sanguineus* s.l. | Vegetation | *Rickettsia +* | 100% *Rhipicephalus sanguineus* (PV018280, Rhsan16SG3) |
| Rhsan6 | *Rh. sanguineus* s.l. | Vegetation | *Rickettsia -* | 100% *Rhipicephalus sanguineus* (PV018281, Rhsan16SG1) |
| Rhsan11 | *Rh. sanguineus* s.l. | Vegetation | *Rickettsia -* | 100% *Rhipicephalus sanguineus* (PV018282, Rhsan16SG1) |
| Rhsan14 | *Rh. sanguineus* s.l. | Vegetation | *Rickettsia -* | 100% *Rhipicephalus sanguineus* (PV018283, Rhsan16SG1) |
| Rhsan22 | *Rh. sanguineus* s.l. | Vegetation | *Rickettsia -* | 99.6% *Rhipicephalus sanguineus* (PV018284, Rhsan16SG4) |
| Rhsan23 | *Rh. sanguineus* s.l. | Vegetation | *Rickettsia -* | 100% *Rhipicephalus sanguineus* (PV018285, Rhsan16SG3) |
| Rhsan29 | *Rh. sanguineus* s.l. | Vegetation | *Rickettsia -* | 100% *Rhipicephalus sanguineus* (PV018286, Rhsan16SG1) |
| Rhsan35 | *Rh. sanguineus* s.l. | Vegetation | *Rickettsia -* | 100% *Rhipicephalus sanguineus* (PV018287, Rhsan16SG1) |
| Rhsan55 | *Rh. sanguineus* s.l. | Vegetation | *Rickettsia -* | 100% *Rhipicephalus sanguineus* (PV018288, Rhsan16SG3) |
| Rhsan64 | *Rh. sanguineus* s.l. | Vegetation | *Rickettsia -* | 100% *Rhipicephalus sanguineus* (PV018289, Rhsan16SG1) |
| Rhsan65 | *Rh. sanguineus* s.l. | Vegetation | *Rickettsia -* | 100% *Rhipicephalus sanguineus* (PV018290, Rhsan16SG1) |
| Rhsan73 | *Rh. sanguineus* s.l. | Vegetation | *Rickettsia -* | 100% *Rhipicephalus sanguineus* (PV018291, Rhsan16SG1 |
| Rhsan76 | *Rh. sanguineus* s.l. | Vegetation | *Rickettsia -* | 100% *Rhipicephalus sanguineus* (PV018292, Rhsan16SG1) |

**Supplementary file 4**: Designation, information on the origins, infection status by *Rickettsia* spp., and mitochondrial 16S rRNA genotypes of remaining Tunisian isolates of *Rhipicephalus rutilus* and *Rh. sanguineus* ticks

Abbreviations: *Rickettsia* (+/-): Positive or negative to *Rickettsia* spp. *ompB* PCR, ^1^ BLAST analysis for mitochondrial 16S rRNA partial sequence of ticks; ^2^ GenBank accession number.
