# Supplementary material for: Molecular identification, genotyping and phylogenetic analysis of Ixodes and Rhipicephalus ticks and their associated spotted fever group Rickettsia species from a single location in northern Tunisia
Source: Front Microbiol. 2025 Aug 14;16:1644524. doi: 10.3389/fmicb.2025.1644524 (PMC12391194; doi:10.3389/fmicb.2025.1644524)
Supplement: Supplementary file 5 [file Table_5.docx]

16S rRNA ticks (GenBank PV029620-PV029653)

SUB15036450 Ixric4-16SrRNA PV018108

SUB15036450 Ixric8-16SrRNA PV018109

SUB15036450 Ixric133-16SrRNA PV018110

SUB15036450 Ixric20-16SrRNA PV018111

SUB15036450 Ixic169-16SrRNA PV018112

SUB15036450 Ixric170-16SrRNA PV018113

SUB15036450 Ixric92-16SrRNA PV018114

SUB15036450 Ixric123-16SrRNA PV018115

SUB15036450 Ixric11-16SrRNA PV018116

SUB15036450 Ixric13-16SrRNA PV018117

SUB15036450 Ixric22-16SrRNA PV018118

SUB15036450 Ixric23-16SrRNA PV018119

SUB15036450 Ixric42-16SrRNA PV018120

SUB15036450 Ixric50-16SrRNA PV018121

SUB15036450 Ixric74-16SrRNA PV018122

SUB15036450 Ixric76-16SrRNA PV018123

SUB15036450 Ixric102-16SrRNA PV018124

SUB15036450 Ixric104-16SrRNA PV018125

SUB15036450 Ixric121-16SrRNA PV018126

SUB15036450 Ixric141-16SrRNA PV018127

SUB15036450 Ixric172-16SrRNA PV018128

SUB15036450 Ixric37-16SrRNA PV018129

SUB15036450 Ixric39-16SrRNA PV018130

SUB15036450 Ixric33-16SrRNA PV018131

SUB15036450 Ixric34-16SrRNA PV018132

SUB15036450 Ixric75-16SrRNA PV018133

SUB15036450 Ixric87-16SrRNA PV018134

SUB15036450 Ixric96-16SrRNA PV018135

SUB15036450 Ixric98-16SrRNA PV018136

SUB15036450 Ixric166-16SrRNA PV018137

SUB15036450 Ixric68-16SrRNA PV018138

SUB15036450 Ixric88-16SrRNA PV018139

SUB15036450 Ixric100-16SrRNA PV018140

SUB15036450 Ixric157-16SrRNA PV018141

SUB15036450 Ixric16-16SrRNA PV018142

SUB15036450 Ixric17-16SrRNA PV018143

SUB15036450 Ixric32-16SrRNA PV018144

SUB15036450 Ixric81-16SrRNA PV018145

SUB15036450 Ixric113-16SrRNA PV018146

SUB15036450 Ixric115-16SrRNA PV018147

SUB15036450 Ixric119-16SrRNA PV018148

SUB15036450 Ixric128-16SrRNA PV018149

SUB15036450 Ixric139-16SrRNA PV018150

SUB15036450 Ixric152-16SrRNA PV018151

SUB15036450 Ixric163-16SrRNA PV018152

SUB15036450 Ixinp24-16SrRNA PV018153

SUB15036450 Ixinp25-16SrRNA PV018154

SUB15036450 Ixinp35-16SrRNA PV018155

SUB15036450 Ixinp43-16SrRNA PV018156

SUB15036450 Ixinp124-16SrRNA PV018157

SUB15036450 Ixinp158-16SrRNA PV018158

SUB15036450 Ixinp171-16SrRNA PV018159

SUB15036450 Ixinp6-16SrRNA PV018160

SUB15036450 Ixinp85-16SrRNA PV018161

SUB15036450 Ixinp131-16SrRNA PV018162

SUB15036450 Ixinp155-16SrRNA PV018163

SUB15036450 Ixinp174-16SrRNA PV018164

SUB15036450 Ixinp3-16SrRNA PV018165

SUB15036450 Ixinp7-16SrRNA PV018166

SUB15036450 Ixinp10-16SrRNA PV018167

SUB15036450 Ixinp12-16SrRNA PV018168

SUB15036450 Ixinp18-16SrRNA PV018169

SUB15036450 Ixinp27-16SrRNA PV018170

SUB15036450 Ixinp29-16SrRNA PV018171

SUB15036450 Ixinp31-16SrRNA PV018172

SUB15036450 Ixinp36-16SrRNA PV018173

SUB15036450 Ixinp38-16SrRNA PV018174

SUB15036450 Ixinp45-16SrRNA PV018175

SUB15036450 Ixinp48-16SrRNA PV018176

SUB15036450 Ixinp51-16SrRNA PV018177

SUB15036450 Ixinp70-16SrRNA PV018178

SUB15036450 Ixinp71-16SrRNA PV018179

SUB15036450 Ixinp72-16SrRNA PV018180

SUB15036450 Ixinp77-16SrRNA PV018181

SUB15036450 Ixinp78-16SrRNA PV018182

SUB15036450 Ixinp79-16SrRNA PV018183

SUB15036450 Ixinp82-16SrRNA PV018184

SUB15036450 Ixinp83-16SrRNA PV018185

SUB15036450 Ixinp84-16SrRNA PV018186

SUB15036450 Ixinp86-16SrRNA PV018187

SUB15036450 Ixinp91-16SrRNA PV018188

SUB15036450 Ixinp93-16SrRNA PV018189

SUB15036450 Ixinp95-16SrRNA PV018190

SUB15036450 Ixinp101-16SrRNA PV018191

SUB15036450 Ixinp162-16SrRNA PV018192

SUB15036450 Ixinp165-16SrRNA PV018193

SUB15036450 Ixinp167-16SrRNA PV018194

SUB15036450 Ixinp175-16SrRNA PV018195

SUB15036450 Ixinp49-16SrRNA PV018196

SUB15036450 Ixinp67-16SrRNA PV018197

SUB15036450 Ixinp89-16SrRNA PV018198

SUB15036450 Ixinp94-16SrRNA PV018199

SUB15036450 Ixinp106-16SrRNA PV018200

SUB15036450 Ixinp107-16SrRNA PV018201

SUB15036450 Ixinp161-16SrRNA PV018202

SUB15036450 Ixinp5-16SrRNA PV018203

SUB15036450 Ixinp46-16SrRNA PV018204

SUB15036450 Ixinp52-16SrRNA PV018205

SUB15036450 Ixinp99-16SrRNA PV018206

SUB15036450 Ixinp129-16SrRNA PV018207

SUB15036450 Ixinp44-16SrRNA PV018208

SUB15036450 Ixinp47-16SrRNA PV018209

SUB15036450 Ixinp73-16SrRNA PV018210

SUB15036450 Ixinp105-16SrRNA PV018211

SUB15036450 Ixinp26-16SrRNA PV018212

SUB15036450 Ixinp1-16SrRNA PV018213

SUB15036450 Ixinp2-16SrRNA PV018214

SUB15036450 Ixinp9-16SrRNA PV018215

SUB15036450 Ixinp14-16SrRNA PV018216

SUB15036450 Ixinp19-16SrRNA PV018217

SUB15036450 Ixinp28-16SrRNA PV018218

SUB15036450 Ixinp40-16SrRNA PV018219

SUB15036450 Ixinp41-16SrRNA PV018220

SUB15036450 Ixinp53-16SrRNA PV018221

SUB15036450 Ixinp69-16SrRNA PV018222

SUB15036450 Ixinp80-16SrRNA PV018223

SUB15036450 Ixinp97-16SrRNA PV018224

SUB15036450 Ixinp103-16SrRNA PV018225

SUB15036450 Ixinp114-16SrRNA PV018226

SUB15036450 Ixinp120-16SrRNA PV018227

SUB15036450 Ixinp122-16SrRNA PV018228

SUB15036450 Ixinp126-16SrRNA PV018229

SUB15036450 Ixinp127-16SrRNA PV018230

SUB15036450 Ixinp132-16SrRNA PV018231

SUB15036450 Ixinp134-16SrRNA PV018232

SUB15036450 Ixinp154-16SrRNA PV018233

SUB15036450 Ixinp156-16SrRNA PV018234

SUB15036450 Ixinp160-16SrRNA PV018235

SUB15036450 Ixinp164-16SrRNA PV018236

SUB15036450 Ixinp173-16SrRNA PV018237

SUB15036450 Ixinp176-16SrRNA PV018238

SUB15036450 Ixinp177-16SrRNA PV018239

SUB15036450 Ixinp168-16SrRNA PV018240

SUB15036450 Ixhex108-16SrRNA PV018241

SUB15036450 Ixhex109-16SrRNA PV018242

SUB15036450 Ixhex111-16SrRNA PV018243

SUB15036450 Ixhex112-16SrRNA PV018244

SUB15036450 Ixhex116-16SrRNA PV018245

SUB15036450 Ixhex117-16SrRNA PV018246

SUB15036450 Ixhex118-16SrRNA PV018247

SUB15036450 Ixhex135-16SrRNA PV018248

SUB15036450 Ixhex136-16SrRNA PV018249

SUB15036450 Ixhex137-16SrRNA PV018250

SUB15036450 Ixhex138-16SrRNA PV018251

SUB15036450 Ixhex140-16SrRNA PV018252

SUB15036450 Ixhex142-16SrRNA PV018253

SUB15036450 Ixhex143-16SrRNA PV018254

SUB15036450 Ixhex144-16SrRNA PV018255

SUB15036450 Ixhex145-16SrRNA PV018256

SUB15036450 Ixhex146-16SrRNA PV018257

SUB15036450 Ixhex147-16SrRNA PV018258

SUB15036450 Ixhex148-16SrRNA PV018259

SUB15036450 Ixhex149-16SrRNA PV018260

SUB15036450 Ixhex150-16SrRNA PV018261

SUB15036450 Ixhex151-16SrRNA PV018262

SUB15036450 Ixhex153-16SrRNA PV018263

SUB15036450 Ixhex185-16SrRNA PV018264

SUB15036450 Rhrut2-16SrRNA PV018265

SUB15036450 Rhrut27-16SrRNA PV018266

SUB15036450 Rhrut15-16SrRNA PV018267

SUB15036450 Rhrut4-16SrRNA PV018268

SUB15036450 Rhrut75-16SrRNA PV018269

SUB15036450 Rhsan13-16SrRNA PV018270

SUB15036450 Rhsan9-16SrRNA PV018271

SUB15036450 Rhsan28-16SrRNA PV018272

SUB15036450 Rhsan20-16SrRNA PV018273

SUB15036450 Rhsan34-16SrRNA PV018274

SUB15036450 Rhsan8-16SrRNA PV018275

SUB15036450 Rhsan12-16SrRNA PV018276

SUB15036450 Rhsan3-16SrRNA PV018277

SUB15036450 Rhsan5-16SrRNA PV018278

SUB15036450 Rhsan60-16SrRNA PV018279

SUB15036450 Rhsan61-16SrRNA PV018280

SUB15036450 Rhsan6-16SrRNA PV018281

SUB15036450 Rhsan11-16SrRNA PV018282

SUB15036450 Rhsan14-16SrRNA PV018283

SUB15036450 Rhsan22-16SrRNA PV018284

SUB15036450 Rhsan23-16SrRNA PV018285

SUB15036450 Rhsan29-16SrRNA PV018286

SUB15036450 Rhsan35-16SrRNA PV018287

SUB15036450 Rhsan55-16SrRNA PV018288

SUB15036450 Rhsan64-16SrRNA PV018289

SUB15036450 Rhsan65-16SrRNA PV018290

SUB15036450 Rhsan73-16SrRNA PV018291

SUB15036450 Rhsan76-16SrRNA PV018292

ompB (GenBank PV029551-PV029619)

BankIt2918931 Ixric4-ompB      PV029551

BankIt2918931 Ixric8-ompB      PV029552

BankIt2918931 Ixric133-ompB    PV029553

BankIt2918931 Ixric20-ompB     PV029554

BankIt2918931 Ixic169-ompB     PV029555

BankIt2918931 Ixric170-ompB    PV029556

BankIt2918931 Ixric123-ompB    PV029557

BankIt2918931 Ixric22-ompB     PV029558

BankIt2918931 Ixric23-ompB     PV029559

BankIt2918931 Ixric42-ompB     PV029560

BankIt2918931 Ixric50-ompB     PV029561

BankIt2918931 Ixric74-ompB     PV029562

BankIt2918931 Ixric76-ompB     PV029563

BankIt2918931 Ixric102-ompB    PV029564

BankIt2918931 Ixinp24-ompB     PV029565

BankIt2918931 Ixinp25-ompB     PV029566

BankIt2918931 Ixinp35-ompB     PV029567

BankIt2918931 Ixinp43-ompB     PV029568

BankIt2918931 Ixinp124-ompB    PV029569

BankIt2918931 Ixinp158-ompB    PV029570

BankIt2918931 Ixinp171-ompB    PV029571

BankIt2918931 Ixinp85-ompB     PV029572

BankIt2918931 Ixinp131-ompB    PV029573

BankIt2918931 Ixinp3-ompB      PV029574

BankIt2918931 Ixinp7-ompB      PV029575

BankIt2918931 Ixinp12-ompB     PV029576

BankIt2918931 Ixinp18-ompB     PV029577

BankIt2918931 Ixinp27-ompB     PV029578

BankIt2918931 Ixinp29-ompB     PV029579

BankIt2918931 Ixinp31-ompB     PV029580

BankIt2918931 Ixinp36-ompB     PV029581

BankIt2918931 Ixinp48-ompB     PV029582

BankIt2918931 Ixinp51-ompB     PV029583

BankIt2918931 Ixinp70-ompB     PV029584

BankIt2918931 Ixinp72-ompB     PV029585

BankIt2918931 Ixinp77-ompB     PV029586

BankIt2918931 Ixinp78-ompB     PV029587

BankIt2918931 Ixinp79-ompB     PV029588

BankIt2918931 Ixinp82-ompB     PV029589

BankIt2918931 Ixinp83-ompB     PV029590

BankIt2918931 Ixinp84-ompB     PV029591

BankIt2918931 Ixinp86-ompB     PV029592

BankIt2918931 Ixinp91-ompB     PV029593

BankIt2918931 Ixinp93-ompB     PV029594

BankIt2918931 Ixinp95-ompB     PV029595

BankIt2918931 Ixinp101-ompB    PV029596

BankIt2918931 Ixinp165-ompB    PV029597

BankIt2918931 Ixinp167-ompB    PV029598

BankIt2918931 Ixinp175-ompB    PV029599

BankIt2918931 Ixric92-ompB     PV029600

BankIt2918931 Ixric11-ompB     PV029601

BankIt2918931 Ixric13-ompB     PV029602

BankIt2918931 Ixric104-ompB    PV029603

BankIt2918931 Ixric121-ompB    PV029604

BankIt2918931 Ixric141-ompB    PV029605

BankIt2918931 Ixinp6-ompB      PV029606

BankIt2918931 Ixinp10-ompB     PV029607

BankIt2918931 Ixinp38-ompB     PV029608

BankIt2918931 Ixinp45-ompB     PV029609

BankIt2918931 Ixinp71-ompB     PV029610

BankIt2918931 Ixinp162-ompB    PV029611

BankIt2918931 Rhrut2-ompB      PV029612

BankIt2918931 Rhrut27-ompB     PV029613

BankIt2918931 Rhrut15-ompB     PV029614

BankIt2918931 Rhsan13-ompB     PV029615

BankIt2918931 Rhsan9-ompB      PV029616

BankIt2918931 Rhsan28-ompB     PV029617

BankIt2918931 Rhsan20-ompB     PV029618

BankIt2918931 Rhsan34-ompB     PV029619

ompA (GenBank PV032631-PV032663)

BankIt2919697 Ixric4-ompA      PV032631

BankIt2919697 Ixric8-ompA      PV032632

BankIt2919697 Ixric133-ompA    PV032633

BankIt2919697 Ixric20-ompA     PV032634

BankIt2919697 Ixic169-ompA     PV032635

BankIt2919697 Ixric170-ompA    PV032636

BankIt2919697 Ixric172-ompA    PV032637

BankIt2919697 Ixric37-ompA     PV032638

BankIt2919697 Ixric39-ompA     PV032639

BankIt2919697 Ixinp24-ompA     PV032640

BankIt2919697 Ixinp25-ompA     PV032641

BankIt2919697 Ixinp35-ompA     PV032642

BankIt2919697 Ixinp43-ompA     PV032643

BankIt2919697 Ixinp124-ompA    PV032644

BankIt2919697 Ixinp158-ompA    PV032645

BankIt2919697 Ixinp171-ompA    PV032646

BankIt2919697 Ixinp155-ompA    PV032647

BankIt2919697 Ixinp174-ompA    PV032648

BankIt2919697 Ixinp49-ompA     PV032649

BankIt2919697 Ixinp67-ompA     PV032650

BankIt2919697 Ixinp89-ompA     PV032651

BankIt2919697 Ixinp94-ompA     PV032652

BankIt2919697 Ixinp106-ompA    PV032653

BankIt2919697 Ixinp107-ompA    PV032654

BankIt2919697 Ixinp161-ompA    PV032655

BankIt2919697 Rhrut2-ompA      PV032656

BankIt2919697 Rhrut27-ompA     PV032657

BankIt2919697 Rhsan13-ompA     PV032658

BankIt2919697 Rhsan9-ompA      PV032659

BankIt2919697 Rhsan28-ompA     PV032660

BankIt2919697 Rhsan8-ompA      PV032661

BankIt2919697 Rhsan12-ompA     PV032662

BankIt2919697 Rhsan16-ompA     PV032663

gltA (GenBank PV029620-PV029653)

BankIt2918945 Ixric4-gltA      PV029620

BankIt2918945 Ixric8-gltA      PV029621

BankIt2918945 Ixric133-gltA    PV029622

BankIt2918945 Ixric123-gltA    PV029623

BankIt2918945 Ixric33-gltA     PV029624

BankIt2918945 Ixric34-gltA     PV029625

BankIt2918945 Ixric75-gltA     PV029626

BankIt2918945 Ixric98-gltA     PV029627

BankIt2918945 Ixinp24-gltA     PV029628

BankIt2918945 Ixinp25-gltA     PV029629

BankIt2918945 Ixinp35-gltA     PV029630

BankIt2918945 Ixinp43-gltA     PV029631

BankIt2918945 Ixinp124-gltA    PV029632

BankIt2918945 Ixinp131-gltA    PV029633

BankIt2918945 Ixinp155-gltA    PV029634

BankIt2918945 Ixinp174-gltA    PV029635

BankIt2918945 Ixinp99-gltA     PV029636

BankIt2918945 Ixinp129-gltA    PV029637

BankIt2918945 Ixric92-gltA     PV029638

BankIt2918945 Ixric87-gltA     PV029639

BankIt2918945 Ixric96-gltA     PV029640

BankIt2918945 Ixric166-gltA    PV029641

BankIt2918945 Ixinp6-gltA      PV029642

BankIt2918945 Ixinp85-gltA     PV029643

BankIt2918945 Ixinp5-gltA      PV029644

BankIt2918945 Ixinp46-gltA     PV029645

BankIt2918945 Ixinp52-gltA     PV029646

BankIt2918945 Rhrut2-gltA      PV029647

BankIt2918945 Rhrut4-gltA      PV029648

BankIt2918945 Rhsan13-gltA     PV029649

BankIt2918945 Rhsan20-gltA     PV029650

BankIt2918945 Rhsan3-gltA      PV029651

BankIt2918945 Rhsan24-gltA     PV029652

BankIt2918945 Rhsan51-gltA     PV029653
