# Supplementary figures and images for: Molecular identification, genotyping and phylogenetic analysis of Ixodes and Rhipicephalus ticks and their associated spotted fever group Rickettsia species from a single location in northern Tunisia
Source: Front Microbiol. 2025 Aug 14;16:1644524. doi: 10.3389/fmicb.2025.1644524 (PMC12391194; doi:10.3389/fmicb.2025.1644524)

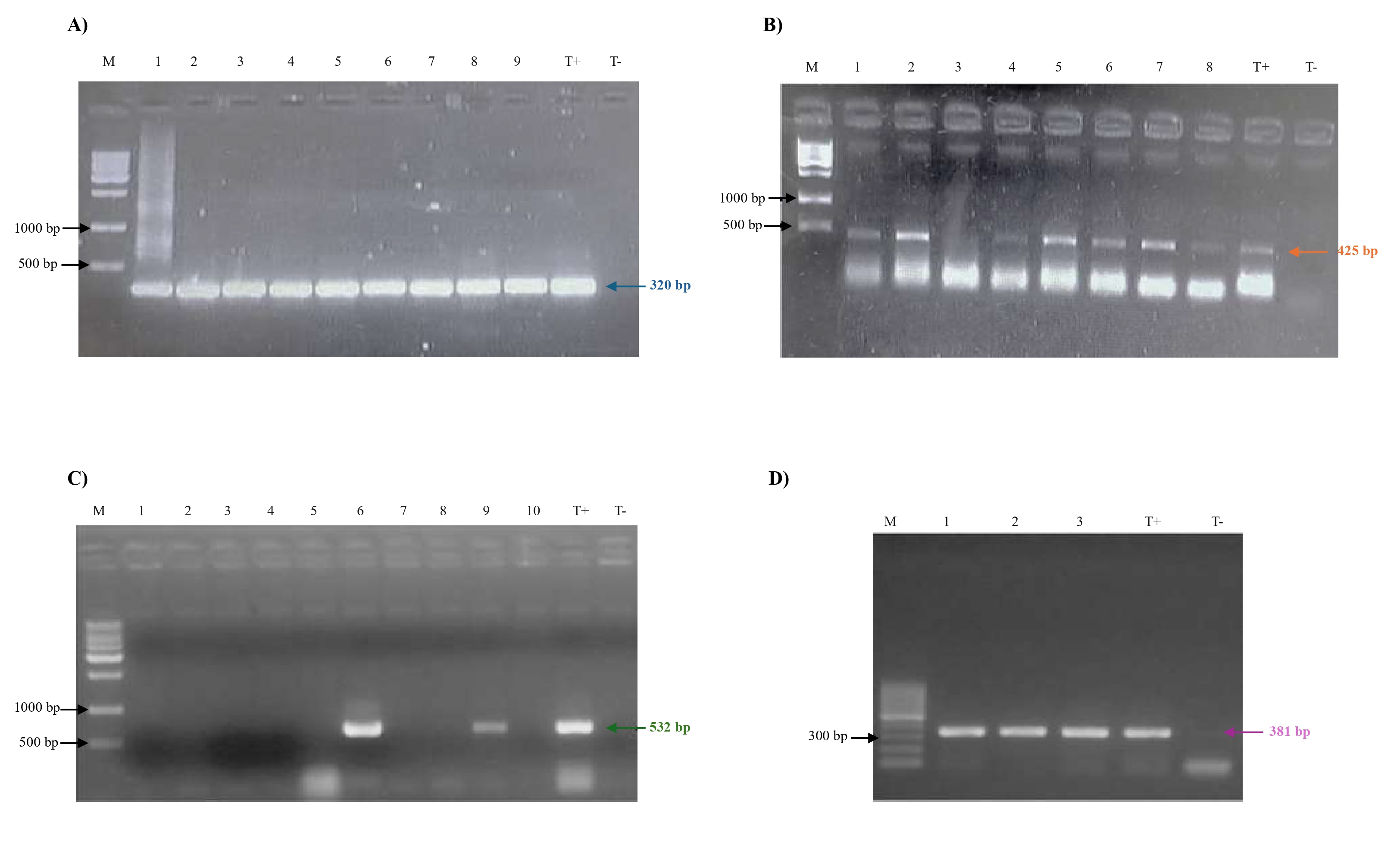

Supplement: Supplementary file 6 [file Image_1.jpeg]
